# Characterising Cancer Cell Responses to Cyclic Hypoxia Using Mathematical Modelling

**DOI:** 10.1007/s11538-024-01359-0

**Published:** 2024-11-06

**Authors:** Giulia L. Celora, Ruby Nixson, Joe M. Pitt-Francis, Philip K. Maini, Helen M. Byrne

**Affiliations:** 1 Department of Mathematics, University College London, Gordon Street, London, 100190 UK; 2 Mathematical Institute, University of Oxford, Andrew Wiles Building, Woodstock Rd, Oxford, OX2 6GG UK; 3 Department of Computer Science, University of Oxford, Parks Rd, Oxford, OX1 3QD UK; 4 Ludwig Institute for Cancer Research, Nuffield Department of Medicine, University of Oxford, Oxford, OX3 7DQ UK

**Keywords:** Tumour Hypoxia, Fluctuating oxygen levels, Cell cycle, Damage repair, Individual-based modelling, Mathematical oncology

## Abstract

In vivo observations show that oxygen levels in tumours can fluctuate on fast and slow timescales. As a result, cancer cells can be periodically exposed to pathologically low oxygen levels; a phenomenon known as cyclic hypoxia. Yet, little is known about the response and adaptation of cancer cells to cyclic, rather than, constant hypoxia. Further, existing in vitro models of cyclic hypoxia fail to capture the complex and heterogeneous oxygen dynamics of tumours growing in vivo. Mathematical models can help to overcome current experimental limitations and, in so doing, offer new insights into the biology of tumour cyclic hypoxia by predicting cell responses to a wide range of cyclic dynamics. We develop an individual-based model to investigate how cell cycle progression and cell fate determination of cancer cells are altered following exposure to cyclic hypoxia. Our model can simulate standard in vitro experiments, such as clonogenic assays and cell cycle experiments, allowing for efficient screening of cell responses under a wide range of cyclic hypoxia conditions. Simulation results show that the same cell line can exhibit markedly different responses to cyclic hypoxia depending on the dynamics of the oxygen fluctuations. We also use our model to investigate the impact of changes to cell cycle checkpoint activation and damage repair on cell responses to cyclic hypoxia. Our simulations suggest that cyclic hypoxia can promote heterogeneity in cellular damage repair activity within vascular tumours.

## Introduction

Uncontrolled proliferation is one of the hallmarks of cancer (Hanahan 2022). However, experimental evidence shows that intra-tumour heterogeneity in proliferation activity is a leading cause of treatment failure, with small numbers of quiescent (i.e., non-proliferative) cancer cells driving drug resistance and relapse (Aguirre-Ghiso 2007; Tomasin and Bruni-Cardoso 2022). This observation highlights the need to understand what environmental and subcellular signals regulate quiescence in cancer (Tomasin and Bruni-Cardoso 2022).

The mitotic cell cycle is commonly divided into four phases: G1 (growth in preparation for DNA replication), S (DNA synthesis), G2 (growth and preparation for mitosis) and M (mitosis). As cells proceed through the cell cycle there are two key decisions to make: whether to initiate DNA replication and whether to undergo mitosis. These decisions are regulated by integrating multiple cellular signalling pathways and extracellular stimuli. At the cell scale, control mechanisms (or checkpoints) guarantee timely and accurate replication of the genome (in the S phase) and its correct segregation into two daughter cells (in the M phase). At the tissue scale, environmental cues, such as growth factors, nutrient levels and mechanical stress, can favour re-entry into, or arrest of, the mitotic cycle to maintain tissue homeostasis by regulating checkpoint dynamics. To maintain high rates of proliferation, cancer cells must disrupt cell cycle regulation mechanisms designed to prevent the replication of damaged/neoplastic cells. Such behaviour is usually associated with mutations or dysregulation of proteins that control cell cycle checkpoints; specifically, cell cycle control in response to DNA damage and S-phase entry (Matthews et al. 2022). Nonetheless, cells may still benefit from having functioning checkpoints that induce quiescence and enable cancer cell survival under unfavourable conditions.

As a solid tumour develops, excessive cell proliferation leads to an imbalance between oxygen supply and demand, resulting in pathologically low oxygen levels (*i.e.,* hypoxia) at distance from the vasculature. Hypoxia is a known driver of cellular quiescence and has been associated with poor treatment outcomes. As hypoxia is toxic for proliferating cells, particularly those actively synthesising DNA, cells that reside in hypoxic regions may enter a quiescent state (Höckel and Vaupel 2001). By transiently exiting the cell cycle, these cells are able to withstand adverse environmental conditions.

Oxygen levels in vascularised tumours are both spatially and temporally heterogeneous (Kawai et al. 2022; Matsumoto et al. 2010; Saxena and Jolly 2019). As a result, regions in which cells are periodically exposed to hypoxia can arise, a phenomenon known as *cyclic hypoxia*. While constant hypoxia typically affects tumour regions at a significant distance from vessels, cyclic hypoxia is observed both close to, and far from, blood vessels, with periods ranging from seconds to days (Bader et al. 2021a; Ron et al. 2019). High-frequency fluctuations are usually associated with vasomotor activity, while vascular remodelling and treatment can generate cycles with longer periods (Michiels et al. 2016). Recent work suggests that self-sustained fluctuations in blood flow might be related to the topology of blood vessel networks, which is known to be abnormal in tumours (Ben-Ami et al. 2022).

Despite experimental evidence suggesting the potential role of cyclic hypoxia in driving tumour aggressiveness, relatively little is known about how cancer cells respond to fluctuating, rather than constant, oxygen levels to promote tumour growth, invasion and metastasis (see Saxena and Jolly 2019 for a recent review). This is because of the several experimental challenges associated with quantifying cyclic hypoxia in vivo and with developing in vitro models that replicate the oxygen dynamics experienced by tumours growing in vivo. Mathematical modelling provides an efficient tool to explore the role of complex oxygen dynamics across temporal and spatial scales, from intracellular signalling within individual cancer cells to emergent population-level tumour dynamics. For example, mathematical modelling has helped elucidate the crosstalk between cyclic hypoxia and gene expression pattern (Zhang et al. 2014) with a focus on HIF-signalling (Leedale et al. 2014). In previous work (Celora et al. 2022; Celora 2022), we have shown how mathematical modelling can be combined with experimental data to study the impact of short-term exposure to a wide range of cyclic hypoxia protocols on cell cycle progression in the colorectal RKO cancer cell line. Here, we extend our cell cycle model to investigate the long-term impact of time-varying oxygen levels on cancer cell survival and the emergent population growth dynamics. The flexibility of our modelling framework allows us to investigate how cell cycle checkpoint and damage repair signalling influence cancer cells’ adaptation to different forms of cyclic hypoxia. In doing so, we obtain new insight into how cyclic hypoxia may contribute to intra-tumour heterogeneity and treatment resistance by favouring the selection of cancer cells which differ in their ability to repair damage.

The paper is organised as follows. In Sect. 2, we review what is currently known about cell cycle progression and cell survival in different hypoxic environments. In Sect. 3, we present a stochastic, individual-based (IB) model of the cell cycle in hypoxia which aims to capture aspects of the biology presented in Sect. 2. In Sect. 4.1, we validate our model by simulating growth dynamics in constant environmental conditions and comparing model output with experimental observations. In Sect. 4.2, we use our model to study how different fluctuating hypoxic environments affect the growth dynamics (see Sect. 4.2.1) and survival outcomes (see Sect. 4.2.2) of cancer cell populations. In Sect. 4.3, simulations of serial passage assays reveal how alterations to damage repair and cell cycle checkpoint signalling may affect cancer cell responses and adaptation to cyclic hypoxia. In Sect. 5, we explain how our results increase our understanding of how cyclic hypoxia may contribute to tumour heterogeneity by allowing the coexistence of cells with different levels of damage repair capacity. We conclude in Sect. 6 by summarising our results and outlining possible directions for future research.

## Cell (dys-)regulation in Hypoxia

When characterising cell responses to hypoxia, it is important to account for the oxygen concentration to which the cells are exposed. In this study, we use the term “hypoxia” to refer to oxygen levels below $c_H=1\%$ $\hbox {O}_{2}$, which is often referred to as pathological hypoxia (McKeown 2014). In practice, a tumour’s tolerance to oxygen shortages depends on its tissue of origin. As such, this threshold should be viewed as an upper bound, rather than an absolute value (McKeown 2014).

**Fig. 1:**
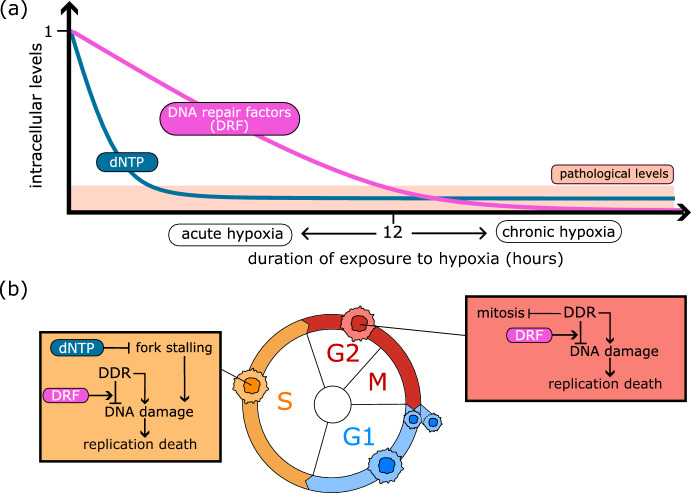
Schematic representation of cell responses as a function of hypoxia duration. **a** Intracellular levels of dNTP quickly drop to pathological levels in hypoxia, determining cell responses to acute hypoxia. Levels of DNA repair factors decrease more slowly than dNTP and, hence, drive cell responses to chronic hypoxia. **b** Cell cycle-specific role of dNTP and DRF levels on the regulation of intracellular mechanisms: DNA synthesis and damage accumulation/repair. Keys: arrow-heads indicate stimulation; bar-heads indicate inhibition; DDR denotes *DNA damage response* and dNTP denotes *deoxynucleotide triphosphates*. More details are given in the main text

The schematic in Fig. 1 summarises how prolonged exposure to hypoxia affects cell physiology by disrupting two fundamental processes: DNA synthesis and repair. The consequences of these perturbations are cell cycle phase specific (see Fig. 1b).

In vitro experiments have shown the rapid reduction in the initiation and progression of DNA synthesis in cells exposed to hypoxia (Foskolou et al. 2017; Pires et al. 2010b). This behaviour has been attributed to impaired functioning of the enzyme ribonucleotide reductase (RNR) (Foskolou et al. 2017; Olcina et al. 2010), which mediates *de novo* production of *deoxynucleotide triphosphates* (dNTPs). Since dNTPs are the building blocks of DNA, the reduction in dNTP levels prevents cells from initiating DNA synthesis (arrest in the G1 phase) and causes DNA synthesis to stall (arrest in the S phase). The stalling of DNA synthesis activates the DNA damage response (DDR), stabilising open replication forks and allowing cells in the S-phase to withstand replication stress. However, exposure to hypoxia also activates an energy-preserving program (Pires et al. 2010a) resulting in reduced production of DNA repair factors (DRF) and, hence, reduced ability to stabilise stalled replication forks. If hypoxic conditions are prolonged (more than 12 hours) arrest in the S phase becomes irreversible and leads eventually to cell death (Pires et al. 2010b; Ng et al. 2018). By contrast, cells that arrest before initiating DNA synthesis can tolerate prolonged exposure to hypoxia since they are not sensitive to replication stress. Differences in the time scales associated with the decreases in levels of dNTPs and DNA repair factors enable cells to distinguish between acute (less than 12 hours) and chronic (more than 12 hours) hypoxia. As a result, cells can adapt their response to oxygen dynamics rather than responding instantaneously to changes in oxygen levels.

If oxygen levels are restored after acute exposure to hypoxia, cells in the S phase can resume DNA synthesis although they may accumulate additional damage during re-oxygenation (Bader et al. 2021b). Depending on the amount of stress/damage sustained, activation of DDR signalling may cause these cells to accumulate in the G2 phase and prevent them from entering mitosis (Bristow and Hill 2008; Goto et al. 2015; Olcina et al. 2010). Damaged cells that successfully repair any damage they have accumulated eventually enter mitosis and replicate; otherwise, they undergo reproductive death (either via activation of the senescence program or via cell death). Regulation of the DDR signalling and damage repair is therefore crucial in determining the long-term impact of hypoxia on cancer cell responses; conversely, hypoxia is known to shift the damage repair capacity of cells (Begg and Tavassoli 2020).

In our previous work (Celora et al. 2022), we focussed on modelling cell responses to acute exposure to constant and cyclic hypoxia. As such, we neglected the role of DNA repair factors and the impact of hypoxia on cell viability. Here, we show how these effects can be included in our framework.

## An Individual-Based Model of in vitro Cancer Cell Dynamics in Hypoxia

### Model Overview

We consider a population of cells that are in a well-mixed (i.e., spatially homogeneous) environment and exposed to externally prescribed, time-varying oxygen levels, $c=c(t)$ [O$_2\%$]. This mimics typical cell culture experiments in oxygen chambers (Kim et al. 2021). For simplicity, we focus on the early stages of population growth, when competition for space and nutrients can be neglected.

**Table 1:** List of the variables characterising a cell (individual) state with a brief description and the range of values that these can take

Variable	Description	Values
$m^{(i)}_\text {dNTP} (t)$	Intracellular dNTP levels in cell *i* at time *t* (a.u.)	[0, 1]
$m^{(i)}_\text {DRF} (t)$	Intracellular DRF levels in cell *i* at time *t* (a.u.)	[0, 1]
$x^{(i)}(t)$	DNA content in cell *i* at time *t*	[1, 2]
$y^{(i)}(t)$	Level of DNA damage in cell *i* at time *t* per unit copy of DNA content (a.u.)	$[0,\infty )$
$z^{(i)}(t)$	Cell cycle state of cell *i* at time *t*	$\left\{ G_1,C_1,S,G_2,C_2\right\}$

The notation (a.u.) stands for arbitrary units

We represent each cell as an individual which can proliferate or die with probabilities that depend on their state. Each cell is characterised by five state variables (see Table 1). The categorical variable *z* indicates the position along the cell cycle (cell cycle state), while the four continuous state variables describe, respectively, DNA content (*x*), damage levels (*y*), intracellular levels of dNTP ($m_\text {dNTP}$) and DNA repair factors ($m_\text {DRF}$). Continuous state variables are included to account for the dynamics of intracellular processes that regulate cell proliferation (via progression through the cell cycle) and death in oxygen-fluctuating environments. Variables *x* and $m_\text {dNTP}$ are introduced to describe the evolution of DNA synthesis in the S phase; variables *y* and $m_\text {DRF}$ are introduced to describe the processes of damage repair.

**Fig. 2:**
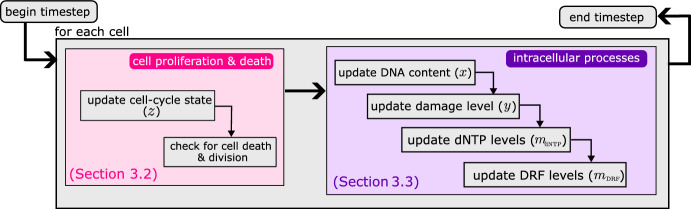
Flowchart illustrating how we implement our stochastic individual-based ( IB) model to simulate in vitro cancer cell dynamics in hypoxia. The algorithm comprises two main subroutines: simulation of cell proliferation and death (pink shaded area); and simulation of intracellular processes (purple shaded area). Details of the implementation are given in Appendix A (Color figure online)

Proliferation, death and state changes of each cancer cell are described by time-discrete stochastic processes. We consider discrete time points: $t_n= n\Delta t\in [0,t_f]$, where $t_f$ is the final time of the simulations and the time-step $\Delta t \in {\mathbb {R}}^+$ is chosen to be sufficiently small to resolve all dynamic processes included in the model. The flow chart in Fig. 2 summarises the procedure used at each time-step to simulate cell fate decisions (i.e., death, division or progression along the cell cycle) and intracellular processes (i.e., DNA replication and damage repair). In the rest of this section we first briefly describe the rules used to simulate cell fate decisions; we then outline the rules used to simulate intracellular processes; namely, DNA synthesis, damage repair and dNTP and DRF production. To conclude, we summarise the simulations we perform and how they are initialised.

### Modelling Cell Proliferation and Death

**Fig. 3:**
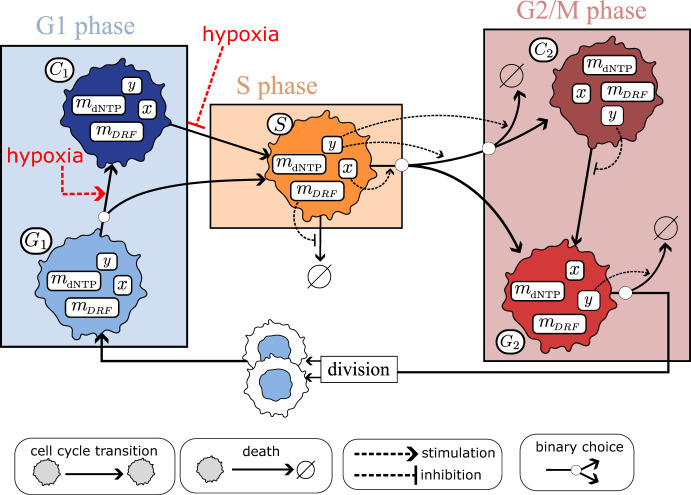
Description of our cell cycle model. Cells can exist in one of 5 cell cycle states ($z\in \left\{ G_1, C_1, S, G_2, C_2\right\}$). Transitions between cell cycle states and cell death depend upon a cell state or oxygen levels as detailed in Appendix A. We illustrate how the internal variables regulate progression through the cell cycle. At the points where the continuous arrows bifurcate, only one of the possible paths is chosen. The symbol $\varnothing$ indicates loss of replication capacity, either via cell death and/or senescence

Following Celora et al. (2022), we assume that cells exist in one of five cell cycle states: $G_1,\ C_1,\ S,\ G_2,\ C_2$. Table 2 summarises the role of each cell cycle state in the model and how they map to (biological) cell cycle phases. At any time-step $t_n$, cells can update their cell cycle state, divide or die with probabilities that depend on the values of their state variables (see Table 1) and oxygen levels as summarised in the schematic in Fig. 3.

**Table 2:** Description of the cell cycle states included in our model and how they map to the standard biological cell cycle phases: G1, S, G2 and M

Cell cycle state (*z*)	Description	Cell cycle phase
$G_1$	Cells preparing to initiate DNA replication	G1
$C_1$	Cells ready to start DNA replication but arrested due to checkpoint activation	G1
*S*	Cells replicating DNA	S
$G_2$	Cells that have completed DNA replication and are preparing for cell division	G2/M
$C_2$	Cells that have completed DNA replication but can not enter mitosis because of checkpoint activation	G2

**Fig. 4:**
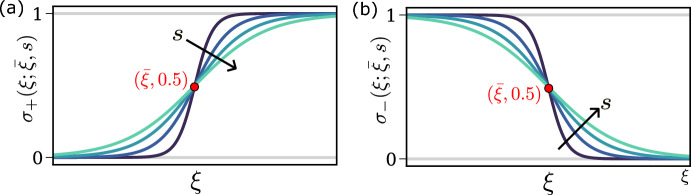
Schematic illustrating how we model the activation (**a**) and inhibition (**b**) of cell cycle transition by a variable $\xi$, which can represent either an internal state variable or externally prescribed oxygen levels The inhibition/activation is modelled using a shifted and rescaled sigmoid function $\sigma$ (see Eq. (1)), parametrised by ${\bar{\xi }}$, i.e., the location of the point of inflection, and *s*, which characterises the steepness of the activation/deactivation curves

We model the stimulatory/inhibitory effects of intracellular and environmental (i.e., oxygen) factors on cell cycle transitions by using the sigmoid function

1 $$\begin{aligned} \sigma _{\pm }(\xi ;{\bar{\xi }},s)=\frac{\exp \left( \pm \frac{\xi -{\bar{\xi }}}{s}\right) }{\exp \left( \pm \frac{\xi -{\bar{\xi }}}{s}\right) +1}, \end{aligned}$$

which is commonly used in modelling non-linear activation responses that are mediated by multistep processes (Ferrell et al. 2011). In Eq. (1) the subscript indicates whether the variable $\xi$ induces a stimulatory ($+$) or inhibitory (−) effect. As shown in Fig. 4, the parameter ${\bar{\xi }}$ shifts the sigmoid function so that its inflection point is located at $\xi ={\bar{\xi }}$, while the parameter *s* regulates the steepness of the sigmoidal curve. For $s\rightarrow 0$, $\sigma _\pm$ converges to a Heaviside step function (switch-like response), while larger values of *s* correspond to a smoother, graded response. Given this formalism, we translate the diagram in Fig. 3 into a set of rules that determine cell death, cell division and how the cell cycle state of each cell is updated from time $t_n$ to time $t_{n+1}$. These rules are detailed in Appendix A.

Cells in the $G_1$ and $C_1$ states are both in the G1 phase; while $G_1$ cells have not committed to entering the S phase, $C_1$ cells have but are transiently arrested due to hypoxia. Transition into, and out of, the $C_1$ state models hypoxia-mediated activation/deactivation of the G1 checkpoint (see Fig. 3). *S* cells remain in this state until they complete DNA replication (i.e., when $x=2$). Cells in the *S* state are sensitive to fork collapse, which occurs when DRF levels drop below a minimal threshold necessary to support the integrity of the DNA replication machinery. Cells in states $G_2$ and $C_2$ are in the G2/M phase. While cells in $G_2$ can attempt mitosis, $C_2$ cells are transiently arrested while they repair accumulated damage (G2 checkpoint). Cell death in the G2/M phase is regulated by a cell damage level, *y*, and can occur either upon transition to the $C_2$ state or via mitotic catastrophe when $G_2$ cells that attempt mitosis detect irreparable damage. Upon division, a $G_2$ cell is replaced by two $G_1$ cells. All values of their internal variables are inherited from the parent cell, except for the DNA content which is split equally between the two daughter cells ($x=1$). Dead/senescent cells are instantaneously removed from the population.

### Modelling Intracellular Processes

We account for the impact of hypoxia on DNA synthesis and intracellular damage dynamics by assuming that changes in $m_\text {dNTP}$ and $m_\text {DRF}$ depend on the externally prescribed oxygen levels *c* (see Fig. 1). As discussed in Sect. 2, we assume that expression levels of dNTP and DRF decrease in hypoxia ($c<c_H$), and increase upon reoxygenation ($c>c_H$). Additional noise in the evolution of dNTP and DRF levels is introduced to account for intercellular heterogeneity. Details on the update rules for $m_\text {dNTP}$ and $m_\text {DRF}$ can be found in Appendix A.3.

#### Modelling DNA Synthesis

The DNA content of cell *i*, $x^{(i)}\in [1,2]$, is constant during the G1 ($x^{(i)}=1$) and G2/M ($x^{(i)}=2$) phases. During the S phase, it increases from $x^{(i)}=1$ to $x^{(i)}=2$ at a rate that is assumed to be proportional to its intracellular levels of dNTPs (*i.e*, $m^{(i)}_\text {dNTP}$). We use the following rule to update the DNA content of cell *i* between times $t_n$ and $t_{n+1}$:

2a $$\begin{aligned} x^{(i)}(t_{n+1})&= \min \left\{ x^{(i)}(t_{n})+\Delta x^{(i)}_n e^{\Theta -\sigma ^2/2},2\right\} , \end{aligned}$$

where $$\Theta \sim {\mathcal {N}}(0,\sigma )$$ and

2b $$\begin{aligned} \Delta x^{(i)}_n={\left\{ \begin{array}{ll} {\bar{v}}_x\, m^{(i)}_\text {dNTP} (t_n) \Delta t,& \quad z^{(i)}=S,\\ 0, & \quad \text{ otherwise }. \end{array}\right. } \end{aligned}$$

**Fig. 5:**
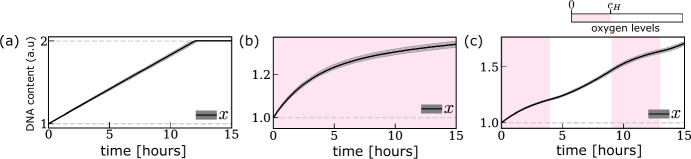
Evolution of DNA content *x*(*t*) in the *S* phase under three different oxygen environments: **a** oxygen-rich environment; **b** chronic hypoxia; **c** (4,5)-cyclic hypoxia. The dark line and the shaded grey area indicate, respectively, the median and 99%-confidence interval for *x*(*t*), measured in arbitrary units (a.u.), and were obtained by simulating Eq. (2) with $z^{(i)}=S$ coupled to Eqs. (4) and (10). The background colours indicate oxygen levels. Parameter values are as indicated in Tables 4 and 5 (Color figure online)

In Eq. (2) the positive constant ${\bar{v}}_x$ [1/hr] represents the maximum rate of DNA synthesis. The random variable $e^\Theta$ is introduced to capture inter-cellular variability in the rate of DNA synthesis due to factors and mechanisms not captured in the model; the choice of a lognormal noise ensures the physical constraint that DNA can not be degraded (i.e., $x^{(i)}(t_{n+1})-x^{(i)}(t_{n})\ge 0$) and the factor $e^{-\sigma ^2/2}$ ensures the noise has mean 1. In Fig. 5, we show simulations of the DNA dynamics in S phase under different oxygen environments obtained by coupling Eq. (2) to the dynamics of $m_\text {dNTP}$, Eq. (10), and oxygen levels, Eq. (4).

#### Modelling Damage Dynamics

We assume that the damage level $y^{(i)}$ of cell *i* increases as a result of replication stress experienced during the S phase (see Sect. 2). Here, *y* is a phenomenological variable that captures the accumulation of different forms of DNA damage associated with hypoxic stress, such as under-replicated regions and single- and double-stranded breaks. Damage is repaired during the S phase and via activation of checkpoint signalling (captured in the model via cells transitioning into the $C_2$ state) in the G2 phase. We assume that in both the S and G2 phases damage repair depends on internal levels of damage repair factors ($m_\text {DRF}$) and that the change in the damage level of cell *i* within a time step $\Delta t$ satisfies:

3a $$\begin{aligned} y^{(i)}(t_{n+1})&=y^{(i)}(t_n)+(1+\Theta )\Delta y^{(i)}, \end{aligned}$$

where $$\Theta \sim {\mathcal {N}}(0,\sigma )$$ and

3b $$\begin{aligned} \Delta y^{(i)}&={\left\{ \begin{array}{ll} \gamma _y\Delta t\,\left[ 1-m^{(i)}_\text {dNTP} (t_n)\right] -{\bar{v}}_y\Delta t\,m^{(i)}_\text {DRF} (t_n)\, y^{(i)}(t_n),& \quad z^{(i)}=S,\\ -{\bar{v}}_y\Delta t\,m^{(i)}_\text {DRF} (t_n)\, y^{(i)}(t_n),& \quad z^{(i)}=C_2,\\ 0, & \quad \text{ otherwise }. \end{array}\right. } \end{aligned}$$

**Fig. 6:**
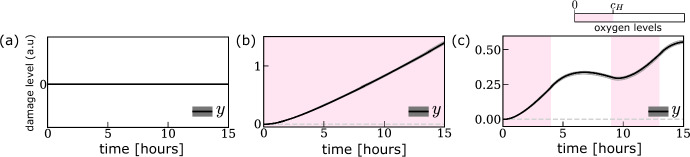
Evolution of damage levels *y*(*t*) in three different oxygen environments: **a** oxygen-rich environment; **b** chronic hypoxia; **c** (4,5)-cyclic hypoxia. The dark line and the shaded grey area indicate, respectively, the median and 99%-confidence interval for *y*(*t*), measured in arbitrary units (a.u.), and were obtained by simulating Eq. (3b) with $z^{(i)}=S$ coupled to Eqs. (4) and (10)-(11). The background colours indicate oxygen levels. Parameter values are as indicated in Tables 4 and 5 (Color figure online)

In Eq. (3b), the positive constants ${\bar{v}}_y$ and $\gamma _y$ represent, respectively, the maximum rate at which damage can be repaired and the rate at which cells accumulate damage to both copies of DNA due to replication stress. For simplicity, we consider only damage induced due to fork stalling at open replication forks, where we assume that damage equally affects the original and copied version of the chromosome so that *y* is directly inheritable. The model could be easily extended to account for asymmetric damage segregation (Xing et al. 2020); we postpone the investigation of this mechanism to future work (see discussion in Sect. 6). As above, we use multiplicative noise to account for intercellular variability in the damage dynamics (see Eq. (3a)). In writing Eq. (3b), we assume that replication stress is proportional to the slowdown in the rate of DNA replication caused by the drop in intracellular dNTP levels (i.e., replication stress $\propto {\bar{v}}_x-\Delta x^{i}_n/\Delta t={\bar{v}}_x/\Delta t (1-m_{\text{ dNTP } }^{(i)})$. Figure 6 shows simulation of how damage levels in S phase evolve under different oxygen environments. Results are obtained by coupling Eq. (3b) to the dynamics of $m_\text {dNTP}$ and $m_\text {DRF}$ (see Eqs. (10)-(11)), and oxygen levels (see Eq. (4)).

### Simulation Results

Numerical simulations of the IB model are performed in Python. More details on the implementation are given in Appendix A; a pseudocode describing how cell proliferation and death, and intracellular processes are simulated is presented in Algorithms 1 and 2.

We use our IB model to simulate the in vitro growth of a population of cancer cells in three oxygen environments:

- *oxygen-rich:*
  4a $$\begin{aligned} c(t)=c_+; \end{aligned}$$
- *chronic hypoxia:*
  4b $$\begin{aligned} c(t)=(c_+-c_-)e^{-\lambda _c t}+c_-; \end{aligned}$$
- *cyclic hypoxia:*
  4c $$\begin{aligned} \begin{aligned} \frac{dc}{dt}&={\left\{ \begin{array}{ll} \lambda _c (c_--c), \quad 0\quad< & t\ (\textrm{mod}\ {\mathcal {T}})\le {\mathcal {T}}_H,\\ \lambda _c (c_+-c), \quad {\mathcal {T}}_H<& t\ (\textrm{mod}\ {\mathcal {T}})\le {\mathcal {T}}, \end{array}\right. }\\ c(0)&=c_+, \end{aligned} \end{aligned}$$
  where $t>0$, the constants $c_{\pm }$ ($c_-<c_H<c_+$) are the minimum and maximum oxygen levels to which cells are exposed, and $\lambda _c$ is the rate at which oxygen levels relax to their equilibrium values. In Eq. (4c), the function $\text{ mod }$ indicates the modulus operator, ${\mathcal {T}}\, [hr]$ is the periodicity of the fluctuations in oxygen levels, and ${\mathcal {T}}_H\, [hr]$ indicates the time of exposure to hypoxia during an oxygen cycle. In other words, cells are repeatedly exposed to ${\mathcal {T}}_H$ hours of hypoxia followed by ${\mathcal {T}}_R={\mathcal {T}}-{\mathcal {T}}_H$ hours of reoxygenation. In what follows, the range of possible cyclic hypoxia protocols are characterised by the tuple $({\mathcal {T}}_H,{\mathcal {T}}_R)$ and the term “$({\mathcal {T}}_H,{\mathcal {T}}_R)$–cyclic hypoxia” refers to the oxygen protocol described by Eq. (4c).

**Fig. 7:**
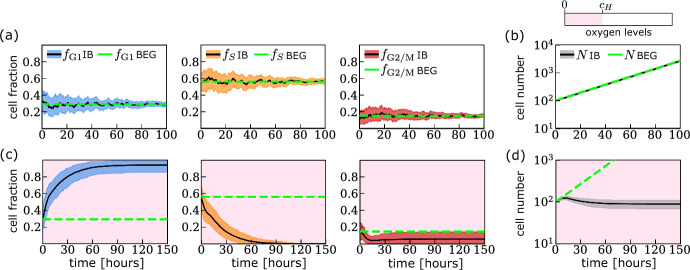
Cell cycle and growth dynamics in constant oxygen environments generated by the IB model. We plot the evolution of the mean and 99%-confidence interval estimates for the evolution of the fraction of cells in each phase of the cell cycle, $f_m$ for $m\in \left\{ \text {G1},\text {S},\text {G2/M}\right\}$, and the total number of cells, *N*, in **a**, **b** oxygen-rich environment and **c**, **d** chronic hypoxia. The dashed green lines indicate the analytical prediction from the balanced exponential growth (BEG) model in oxygen-rich environments (see Sect. 1). The background colour indicates oxygen levels. Parameter values for the BEG and IB model are the same and are as indicated in Tables 3, 4, 5 and 6 (Color figure online)

**Fig. 8:**
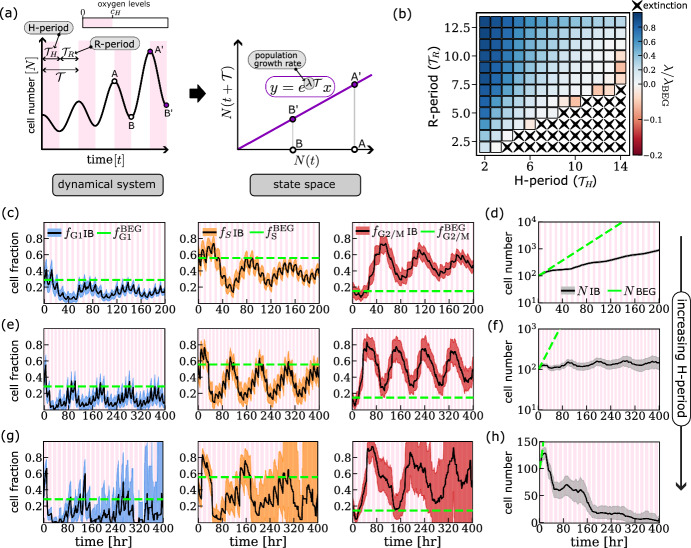
Cell cycle and growth dynamics under different cyclic oxygen environments. **a** Schematic showing how the population growth rate is defined in a fluctuating environment (i.e., $dN/dt=r(t)N$ where *r* is a ${\mathcal {T}}$-periodic function). The time-evolution of cell number *N* deviates from the exponential growth model (see left-hand side plot). However, when projected onto the $(N(t),N(t+{\mathcal {T}}))$ state space, the behaviour is analogous to that of an exponentially growing population in a constant environment (see right-hand side plot), with the population growth rate $\lambda =\int _{0}^{\mathcal {T}}r(\xi )d\xi /{\mathcal {T}}$. **b** Estimated population growth rate $\lambda$ for a range of cyclic hypoxia protocols. Crosses indicate conditions for which the cell population goes extinct with probability $\ge 90\%$. **c**-**h** We plot the evolution of the fraction of cells in each phase of the cell cycle, $f_m$ with $m\in \left\{ \text {G1},\text {S},\text {G2/M}\right\}$, and the total number of cells, *N*, as predicted by the IB model for **(c)**-**(d)** (4,5)–cyclic hypoxia; **(e)**-**(f)** (7,5)–cyclic hypoxia; and **(g)**-**(h)** (11,5)–cyclic hypoxia. Parameter values, variables and colours are as in Fig. 7 (Color figure online)

**Fig. 9:**
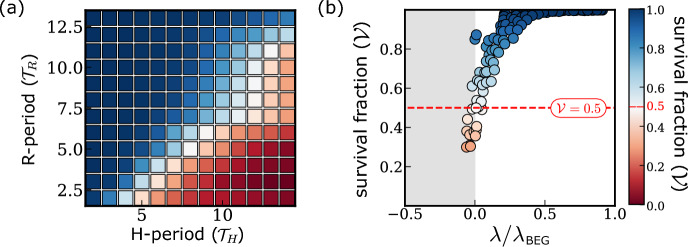
Cell survival in different oxygen environments. **a** Mean estimator for the cell survival fraction ${\mathcal {V}}$ (see Sect. 3.4.2) in a range of cyclic hypoxia conditions. **b** Scatter plot illustrating the relation between the population growth rate $\lambda$ (see Fig. 8b) and cell survival for the cyclic hypoxia conditions studied in **a** except those that lead to extinction of the cell population (see Fig. 8b). Parameters are as indicated in Tables 3, 4, 5 and 6

**Fig. 10:**
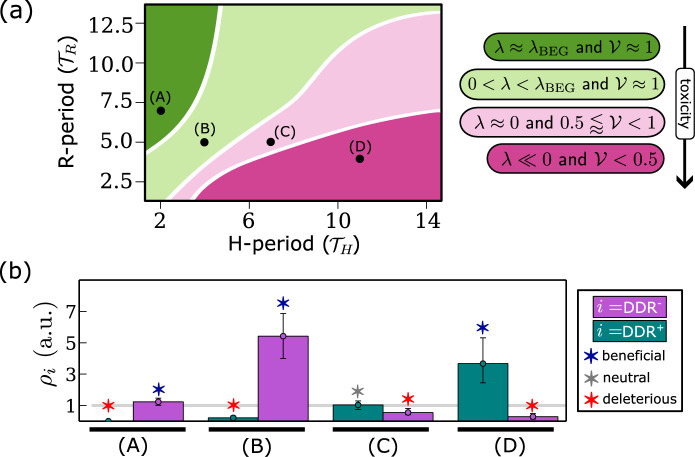
**a** Schematic summarising the characteristic responses of wild-type cells to different cyclic hypoxia protocols. We decompose the $({\mathcal {T}}_H,{\mathcal {T}}_R)$ space into four regions characterised by the estimated values of the population growth rate $\lambda$ (see Fig. 8) and survival fraction ${\mathcal {V}}$ (see Fig. 9). **b** Histograms showing the relative fitness $\rho _{\text {DDR}^\pm }$ (see Eq. (6)) for different cyclic hypoxia protocols: (A) (2,7)–cyclic hypoxia; (B) (4,5)–cyclic hypoxia; (C) (7,5)–cyclic hypoxia; (D) (11,4)–cyclic hypoxia. Red, grey and blue stars indicate DDR alterations that are, respectively, beneficial ($\rho _i>1$, p-value=0.001), deleterious ($\rho _i<1$, *p* value=0.001) or neutral (if neither beneficial or deleterious). More details on how we quantify relative fitness are given in Sect. 3.4.3. Parameter values are as indicated in Tables 3, 4, 5 and 6 (Color figure online)

**Fig. 11:**
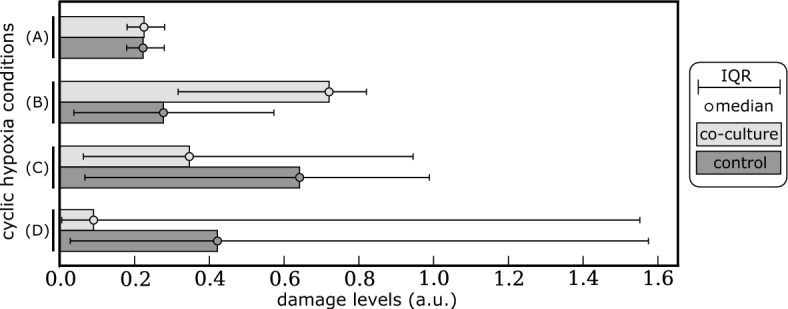
Median $m_D$ and interquartile range IQR$_D$ of the damage distribution (defined in Eq. (7)) for co-culture (light grey) and control (dark grey) conditions for cells grown in the same cyclic hypoxia conditions investigated in Fig. 10: A (2,7)–cyclic hypoxia; B (4,5)–cyclic hypoxia; C (7,5)–cyclic hypoxia; D (11,4)–cyclic hypoxia. Parameters are as indicated in Tables 3, 4, 5 and 6

As standard in in vitro experiments, we initialise cells in a regime of (asynchronous) balanced exponential growth (Celora et al. 2022; Webb 1987) using the procedure outlined in Algorithm 3. This is the equilibrium regime predicted by the model when cells are exposed to oxygen-rich environments (see Sect. 4.2). Unless otherwise stated, simulations are initialised with $n_0=100$ cells. For each numerical experiment and set of parameters, we perform 100 realisations of the IB model and use the obtained data to extract the statistical metrics illustrated in Figs. 7, 8, 9, 10 and 11.

#### Model Parameters

Where possible, model parameters are estimated from the literature, and based on the colorectal RKO cell line which was the focus of previous theoretical (Celora et al. 2022) and experimental studies  (Bader et al. 2021b) on cyclic hypoxia. See Appendix C for futher details (parameter values given in Tables 3, 4 and 5).

We account for cell lines with different regulation of damage repair by varying parameters modulating G2 checkpoint activation (i.e., probability of cell transitioning into and out of the cell cycle state $C_2$) in response to damage, see Eq. (8d); namely, parameters $y^{\text {on}}_{C_2}$, $y^{\text {off}}_{C_2}$ and $s^{\text {on}}_{C_2}$ (see Table 3). We model cells with enhanced damage repair activity ($\hbox {DDR}^{+}$ cells) compared to the reference (or wild-type) behaviour ($\hbox {DDR}^{\textrm{wt}}$ cells) by decreasing $y^{\text {on}}_{C_2}$, $y^{\text {off}}_{C_2}$ and $s^{\text {on}}_{C_2}$ relative to their default values; we account for defective damage repair activity ($\hbox {DDR}^{-}$ cells) compared to wild-type behaviour ($\hbox {DDR}^{\textrm{wt}}$ cells) by increasing $y^{\text {on}}_{C_2}$, $y^{\text {off}}_{C_2}$ and $s^{\text {on}}_{C_2}$ relative to their default values. For more details, see Appendix C.1.

#### Clonogenic Assays

We estimate cancer cell survival in cyclic hypoxia by simulating in silico clonogenic assays following a standard “plating before treatment” approach (Franken et al. 2006). This means that cells are first plated and then exposed to cyclic hypoxia. After being exposed to cyclic hypoxia for $t_R=15\,({\mathcal {T}}_H+{\mathcal {T}}_R)$ hours, cells are cultured in ambient oxygen conditions ($21\% \text{ O}_2$) for 10 days. The survival fraction is estimated at the end of the 10 days as the ratio

5 $$\begin{aligned} {\mathcal {V}}=\frac{\#\text { of colonies formed}}{\#\text { cell initially plated}}. \end{aligned}$$

In Eq. (5) we define a colony as a cluster of at least 50 cells that originate from the same progenitor. Note that, in writing Eq. (5), we follow the standard convention by defining survival as the ability of cells to escape replicative death and maintain uncontrolled proliferation when exposed to toxic agents (here cyclic hypoxia). We remark that replicative death can be due to cell death but also persistent cell cycle arrest.

#### Serial Passage Assays

We estimate the relative fitness of distinct cancer cell lines under different cyclic hypoxia conditions by simulating serial passage assays. We simulate co-cultures of three cell lines; namely $\hbox {DDR}^{+}$, $\hbox {DDR}^{-}$ and $\hbox {DDR}^{\textrm{wt}}$ (modelled by changing parameter values as described in Sect. 3.4.1). We initialise the simulations with 150 cells from each cell line, for a total of 450 cells. While being exposed to a specific cyclic hypoxia protocol, cells are “passaged” every $\lfloor 48/{\mathcal {T}}\rfloor {\mathcal {T}}$ hours (i.e., approximately every 2 days) where $\lfloor \cdot \rfloor$ indicates the floor function. Replating is simulated by randomly sampling 450 cells from the population; oversampling is used if the size of the population at the time of the replating is less than 450. This procedure artificially introduces a carrying capacity and enhances the positive selection pressure on cells that are more adapted to cyclic hypoxia. The fitness of $\hbox {DDR}^\pm$ cells relative to $\hbox {DDR}^{\textrm{wt}}$ cells is quantified by estimating the ratio

6 $$\begin{aligned} \rho _{\text {DDR}^\pm } = \frac{\# \text {DDR}^{\pm }\text { cells}}{\# \text {DDR}^{\textrm{wt}}\ \text {cells}} \end{aligned}$$

after passaging the population 10 times. $\hbox {DDR}^{\pm }$ cells have a fitness advantage in cyclic hypoxia over $\hbox {DDR}^{\textrm{wt}}$ cells if $\rho _{\text {DDR}^\pm }>1$ (and conversely). Since $\rho _{\text {DDR}^\pm }$ are stochastic variables, statistical evidence for the alternative hypothesis $\rho _{\text {DDR}^\pm }\le 1$ and $\rho _{\text {DDR}^\pm }\ge 1$ is tested using a one-sample one-tail t-test with *p* value 0.001.

## Results

We use our individual-based (IB) model to simulate cancer cell responses to different oxygen environments. In Sect. 4.1, we demonstrate that the IB model reproduces the cell cycle and population growth dynamics observed in vitro under constant oxygen-rich and chronically hypoxic conditions. In Sect. 4.2, we simulate cell culture and clonogenic assay experiments in a wide range of cyclic hypoxia environments. We identify a range of population-level dynamics for different cyclic hypoxia protocols: sustained growth, dormancy and population extinction. Using the IB model, we can relate population-level behaviour to the dynamics of individual cell states and specifically their damage regulation. Finally, in Sect. 4.3, we study how damage repair capacity influences cancer cell fitness under different cyclic hypoxia conditions by simulating serial passage assays.

### Model Predictions in Constant Environments

We validate our IB model by simulating the cell cycle and growth dynamics of a population of wild-type cancer cells under constant oxygen conditions. The results are shown in Fig. 7.

As expected, in the oxygen-rich environment (see Figs. 7a, b) the model predicts balanced exponential growth (note that in Fig. 7b we use a log scale for the y-axis). The total number of cells *N* eventually grows exponentially at a constant rate $\lambda _\text {BEG}$, while the fraction of cells in each cell cycle phase asymptotes to an equilibrium value, $f^\text {BEG}_i$ for $i\in \left\{ \text {G1},\text {S},\text {G2/M}\right\}$, with uncertainty in the values of the cell fractions $f_i$ decreasing over time. This is in line with the predictions of the deterministic model in Celora et al. (2022). The relationship between the IB and deterministic models is discussed in Appendix B.

Under constant hypoxia (see Figs. 7c, d), after an initial transient, the average number of cells evolves to a constant value. While the number of cells increases for the first $\approx 12$ hours, it then decreases due to the death of cells in the S phase as a result of fork collapse. At long times, most cells are in the G1 phase where they arrested via activation of the G1 checkpoint, (i.e., they are locked in state $C_1$). The delayed decrease in population size due to the death of cells in the S phase and the predicted accumulation of quiescent cells in chronic hypoxia agree with observations from experiments on the RKO cell line culture (Bader et al. 2021a).

### Characterising the Wild-Type Responses to Different Cyclic Hypoxia Environments

#### Growth Dynamics

We use the IB model to simulate the cell cycle and growth dynamics of a population of cancer cells under a range of cyclic hypoxia conditions. The results presented in Fig. 8 show that the long-term growth dynamics depend on the oxygen protocol used. We characterise these dynamics by estimating the asymptotic population growth rate, $\lambda$ (see Fig. 8b). This is defined by fitting an exponential function to the change in population size over a period ${\mathcal {T}}$ (see schematic in Fig. 8a). Asymptotically, and assuming a sufficiently large population of cells, the estimated $\lambda$ is expected to be independent of the time *t* chosen for its estimation.

When ${\mathcal {T}}_H$ is sufficiently short, the model predicts sustained population growth, albeit at a lower rate than in oxygen-rich conditions (i.e., during the balanced exponential growth regime), i.e., $0<\lambda \lessapprox \lambda _\text {BEG}$; this is the case, for example, when cells are exposed to (4,5)–cyclic hypoxia (see Fig. 8d). When considering the corresponding cell cycle dynamics, despite the persistent fluctuations in the cell cycle fractions, we observe a systematic increase in the fraction of cells in the G2/M phase, $f_\text {G2/M}$ (see Fig. 8f). We note that the period of the fluctuations in $f_\text {G2/M}$ is eventually the same as for the oxygen levels – in this case 9 hours. As ${\mathcal {T}}_H$ increases, the IB model predicts substantial inhibition of population growth (or growth arrest); this is the case, for example, when simulating exposure of cells to (7,5)–cyclic hypoxia (see Fig. 8f). Recall that under constant hypoxia, growth inhibition is due to cells arresting in the G1 phase. By contrast, under (7,5)-cyclic hypoxia, cells continue proceeding through the cell cycle (compare Figs. 7d and 8e) suggesting that cell proliferation continues even though the total number of cells in the population is not increasing. There are two possible causes of population growth arrest (or population dormancy) (Wells et al. 2013): cell cycle arrest or a balance between cell death and proliferation. We conclude that population dormancy in (7,5)–cyclic hypoxia is due to an increase in cell death. Finally, when considering cyclic hypoxia protocols with larger ${\mathcal {T}}_H$, the model predicts population extinction with high likelihood (see crosses in Fig. 8b); this is the case, for example, when simulating exposure to (11,5)–cyclic hypoxia (see Fig. 8h). We note that the uncertainty in model predictions for the cell cycle dynamics increases on the long-time scale. The widening of the confidence intervals in Fig. 8g is due to the increased dominance of demographic noise as the number of cells approaches zero. Nonetheless, all model realisations eventually predict population extinction.

#### Cell Survival

In the simulations presented in Sect. 4.2.1, we investigated the impact of fluctuating oxygen levels on the emergent population growth dynamics. In this section, we investigate cell survival in different oxygen environments by simulating clonogenic assay experiments (see  Sect. 3.4.2).

In Fig. 9a, we report estimated values of the survival fraction ${\mathcal {V}}$ for different cyclic hypoxia protocols. As in Fig. 8, we characterise cyclic hypoxia environments by the hypoxia period, ${\mathcal {T}}_H$, and the reoxygenation period, ${\mathcal {T}}_R$. We find significant variation in the survival fraction as ${\mathcal {T}}_H$ and ${\mathcal {T}}_H$ vary. For sufficiently large reoxygenation periods ${\mathcal {T}}_R$, cells are likely to survive and ${\mathcal {V}}\approx 1$. In contrast, cells are more likely to die than survive when the reoxygenation period is short and the hypoxia period is sufficiently long. Overall, our results highlight that both the overall time of exposure to hypoxia and the evolution of the oxygen dynamics are important in determining the extent to which hypoxia is toxic for cells.

The results presented in Fig. 9b suggest that estimates of cell survival and population growth rates in cyclic hypoxia are related. In all conditions where we predict a positive growth rate, the survival fraction never drops below 0.5 (see red line in Fig. 9b). This suggests that cells must be more likely to survive than to die to avoid population extinction. While this is intuitive when considering a homogeneous population in which the survival probability is the same for all cells, in our model, cell cycle heterogeneity influences a cell’s survival probability (see Appendix D). The correlation between the initial cell cycle distribution and the estimated survival probability is lost when cells are exposed to fluctuating oxygen levels for sufficiently long times. The decay time scale for such correlations depends on the oxygen dynamics and tends to infinity when ${\mathcal {T}}_R\rightarrow 0$ (i.e., under chronic hypoxia). This is because, in our model, G1 checkpoint arrest under chronic hypoxia is irreversible (see Fig. 7c) so that the estimates of cell survival are determined, even at long times, by the initial cell cycle distribution.

Overall, a decrease in cell survival ${\mathcal {V}}$ corresponds to a decrease in the population growth rate $\lambda$. Nonetheless, we identify a significant range of environmental conditions in which the population growth rate $\lambda$ decreases even though ${\mathcal {V}}\approx 1$. In these cases, the reduction in the population growth rate is driven by the activation of cell cycle checkpoints and the consequent increase in cell cycle duration, rather than increased cell death (see, for example, Fig. 7c, d).

### Characterising the Link Between Damage Repair Capacity and Cancer Cell Responses to Cyclic Hypoxia

In the previous section, we showed how the response of a cancer cell line to cyclic hypoxia depends on how the oxygen levels fluctuate (i.e., the values of ${\mathcal {T}}_H$ and ${\mathcal {T}}_R$). Based on these results, we now partition the $({\mathcal {T}}_H,{\mathcal {T}}_R)$ parameter space into four regions depending on the predicted cell responses (see Fig. 10a). As ${\mathcal {T}}_R$ decreases and ${\mathcal {T}}_H$ increases (i.e., transitioning from the dark green to the dark pink regions in Fig. 10a), the environmental conditions become increasingly toxic for cancer cells. Genetic and phenotypic heterogeneity in the regulation of DNA damage response (DDR) and cell-cycle checkpoint signalling has been observed in solid tumours (Begg and Tavassoli 2020; Jiang et al. 2020). This includes: alterations that silence DDR signalling (Jiang et al. 2020) ($\hbox {DDR}^{-}$ cells), thereby allowing cells to proliferate faster by suppressing damage repair signalling; and alterations that enhance DDR signalling (Wu et al. 2023) ($\hbox {DDR}^{+}$ cells), thereby promoting cell repair signalling and survival, and, therefore, resistance to chemo- and radiotherapy. We simulate serial passage assays to investigate how these alterations to damage repair signalling affect cancer cell fitness in different fluctuating oxygen environments (see Sect. 3.4.3).

The results are presented in Fig. 10b. Overall, we find that the estimated relative fitness $\rho$ of both $\hbox {DDR}^{+}$ and $\hbox {DDR}^{-}$ cells depends on the cyclic hypoxia protocols. For $\hbox {DDR}^{+}$ cells, $\rho$ increases as the extent to which cyclic hypoxia is toxic for wild-type cells increases. In contrast, $\rho _{\text {DDR}^-}$ depends non-monotonically on cyclic hypoxia toxicity. Under cyclic hypoxia conditions that are harmless for wild-type cells (dark green region in Fig. 10a; condition (A) in Fig. 10b), enhanced damage repair capacity is deleterious ($\rho _{\text {DDR}^+}<1$), while deficiencies in damage repair capacity are mildly beneficial ($\rho _{\text {DDR}^-} > rapprox 1$). In contrast, under cyclic hypoxia conditions that are highly toxic for wild-type cells (dark pink region in Fig. 10a; condition (D) in Fig. 10b), enhanced activation of the DDR increases cell fitness ($\rho _{\text {DDR}^+}>1$), while deficiencies in the DDR are significantly deleterious for cells. Between these two extremes (i.e., protocols within the light green and light pink regions of the schematic in Fig. 10a; conditions (B) and (C) in Fig. 10b), the model predicts that enhanced damage repair capacity switches from being deleterious to being beneficial, while deleterious damage repair capacity becomes increasingly deleterious. Interestingly, these transitions are characterised by regimes in which the composition of the population at the end of the simulations is highly heterogeneous, with $\hbox {DDR}^{+}$, $\hbox {DDR}^{-}$ and $\hbox {DDR}^{\textrm{wt}}$ cells coexisting even after several passages. For example, in case (C) in Fig. 10b, the coexistence of different cell types is reflected in $\rho _{\text {DDR}^+}$ and $\rho _{\text {DDR}^-}$ being both close to one. This suggests that cyclic hypoxia can give rise to conditions in which the fitness landscape associated with DDR regulation is flat and, consequently, natural selection is very slow.

#### Heterogeneity in Cell Damage Repair Capacity Shapes Damage Distribution Under Cyclic Hypoxia

To better understand the relation between damage repair and cell fitness in cyclic hypoxia, we look at how selection reshapes the damage distribution within the cell population. We quantify this by comparing the cell damage distribution in the co-culture experiment with the distribution under control conditions where serial passage assays are only performed with $\hbox {DDR}^{\textrm{wt}}$ cells (i.e., default values of the model parameters). The use of the control case for comparison is important since cell passaging can alter both the cell cycle and cell damage distributions. In general, because cells are exposed to fluctuating oxygen levels, the damage distribution eventually converges to a periodic function that fluctuates with the same frequency as the passaging, (see Appendix E). The results, therefore, depend on the time *t* at which the damage distribution is computed. Here we focus on the damage distribution at the end of the final hypoxic phase:

7 $$\begin{aligned} f_d(y)={\mathbb {P}}\left( \text {a cell has damage level }y\text { at time } t=(n_{f}-1){\mathcal {T}}+{\mathcal {T}}_H\right) , \quad y\ge 0, \end{aligned}$$

where $n_f=10\lfloor 48/{\mathcal {T}}\rfloor$ indicates the total number of oxygen cycles to which the cells have been exposed during the serial passage assay. This is the time at which selection (via cell passaging) operates.

The results presented in Fig. 11 show that damage levels in the co-culture and control experiments can differ markedly depending on the cyclic hypoxia protocol considered. For (2,7)-cyclic hypoxia, despite the reported mild advantage of $\hbox {DDR}^{-}$ cells (see (A) Fig. 10b), damage levels are comparable between the co-culture and control experiments. The brief exposure to hypoxia is not sufficient to drive significant damage accumulation even in cancer cells with deficiencies in damage repair capacity. When considering (4,5)-cyclic hypoxia, $\hbox {DDR}^{-}$ cells are predicted to have a significant advantage (see (B) Fig. 10b). However, unlike in (2,7)–cyclic hypoxia, accumulation of $\hbox {DDR}^{-}$ cells is associated with the median damage level in co-culture conditions being significantly higher than in the control conditions. In this intermediate regime, deficiencies in damage repair capacity are most beneficial for cancer cells as they promote proliferation in the face of higher, but not lethal, damage/levels. For more toxic cyclic hypoxia conditions (i.e., light and dark pink regions in Fig. 10a), the trend reverses; lower levels of damage are recorded for the co-culture than control conditions (see results for (C) (7,5)-cyclic hypoxia and (D) (11,4)-cyclic hypoxia in Fig. 11). The environmental toxicity is such that there is no benefit in attempting to proliferate; and cells are better off prioritising their survival by enhancing damage repair signalling and, thereby, maintaining low damage levels (see Fig. 10).

## Application of the Result to Intra-Tumour Heterogeneity

Changes to the regulation of cell cycle checkpoints and damage repair pathways are common in cancer as they sustain uncontrolled proliferation (Viner-Breuer et al. 2019; Jiang et al. 2020). However, in conditions where proliferation can not be sustained, functioning checkpoint regulation can play an essential role in favouring cancer cell survival. For example, in tumour regions that are chronically exposed to severe hypoxia, cells experience replication stress and, therefore, activation of cell cycle checkpoints in response to low oxygen levels (i.e., hypoxic stress) can be crucial for cancer cell survival (Qiu et al. 2017; Pires et al. 2010b, a). The different roles of cell cycle checkpoints in well-oxygenated and hypoxic regions influence the regulation of cancer cell proliferation, thus favouring heterogeneity in hypoxic tumours (Begg and Tavassoli 2020; Emami Nejad et al. 2021).

Heterogenous blood flow in vascularised tumours can generate tissues in which oxygen levels fluctuate, exposing cells to cyclic hypoxia. The period and amplitude of such fluctuations may vary with the distance from the closest vessel. In Ardaševa et al. 2020, it was proposed that spatiotemporal variability in oxygen levels creates ecological niches that foster intratumour phenotypic heterogeneity along the cell metabolic axis. Our results suggest that spatiotemporal heterogeneity in tumour oxygen levels can contribute to intratumour heterogeneity in damage repair capacity. This is because the impact of damage repair capacity on cellular fitness under cyclic hypoxia depends on both the frequency and duration of hypoxia periods (see Fig. 10). While deficient damage repair capacity is advantageous for cancer cells when hypoxia periods are rare, it is deleterious when hypoxia periods are long and frequent. Under such environmental conditions, enhanced damage repair capacity is necessary to sustain prolonged checkpoint activation and allow cell survival. Interestingly, we can identify intermediate cyclic hypoxia conditions under which cancer cells with different damage repair capabilities coexist. This suggests that cells with enhanced damage repair capacity, which is usually associated with resistance to treatment, may be located in regions that are primarily hypoxic and highly toxic for cancer cells, and also in regions that are frequently reoxygenated and can sustain cancer cell proliferation, albeit at a lower rate than in better oxygenated areas.

## Conclusion

We have developed a stochastic, individual-based (IB) model of in vitro cancer cell cultures to study the impact of hypoxia-driven cell cycle dysregulation on cancer cell responses (e.g., proliferation, survival and damage regulation) to different dynamic oxygen environments. Our model extends previous work on modelling cell cycle progression in cyclic hypoxia (Celora et al. 2022) by coupling cell cycle progression to damage repair dynamics. Interestingly, we find that cancer cell responses significantly change depending on the dynamics of the oxygen levels to which cells are exposed as well as their damage repair capacity.

Our model describes how oxygen fluctuations impact cell cycle progression and cell survival by affecting DNA replication and repair within cells. In Sect. 4.2, we showed the cell cycle and growth dynamics predicted by the model for different oxygen environments. In constant oxygen environments, the model reproduces the expected population dynamics for non-confluent cell cultures: balanced exponential growth and growth arrest driven by cell quiescence in the G1 phase. Interestingly, we found that, depending on the duration and frequency of hypoxia periods, cyclic hypoxia can yield very different growth patterns in the same cancer cell population: exponential growth, saturated growth or population extinction. By simulating in vitro clonogenic assays, we were further able to characterise cell survival in different cyclic hypoxia conditions. By combining population growth rate and survival estimates, we partitioned the space of possible cyclic hypoxia protocols (i.e., $({\mathcal {T}}_H,{\mathcal {T}}_R)$-space) into four regions, each associated with qualitatively distinct cancer cell responses (see Fig. 10a).

In Sect. 4.3, we studied how alterations to DNA damage response and cell cycle checkpoint signalling (or, in brief, cell damage repair capacity) influence cancer cell responses to cyclic hypoxia. We considered two types of alterations: deficient damage repair capacity ($\hbox {DDR}^{-}$), which promotes uncontrolled proliferation while hindering damage repair; and enhanced damage repair capacity ($\hbox {DDR}^{+}$), which promotes damage repair and cell survival. Our results suggest that cyclic hypoxia may define different environmental niches: those in which either $\hbox {DDR}^{+}$ or $\hbox {DDR}^{-}$ cells localise; and those in which cells with different DDR signalling coexist. We concluded by discussing the predictions of our model in the context of intratumour heterogeneity in vascularised tumours.

There are several ways in which our work could be extended. We developed our IB model to replicate in vitro monolayer conditions to allow for comparison and validation with experimental data. While cell culture experiments are effective for building a mechanistic understanding, they can not capture the complexity of interactions and 3D organisation of tumours growing in vivo. Hence, there is a growing interest in advancing 3D tumour cultures, such as multicellular spheroids and organoids, to bridge the gap between in vitro and in vivo conditions. A natural extension of our work would be to integrate our cell cycle model within a multiscale framework to study spheroid growth, using either IB (Bull et al. 2020; Hamis et al. 2021; Ghaffarizadeh et al. 2018; Jiménez-Sánchez et al. 2021) or continuous modelling (Murphy et al. 2023; Pérez-Aliacar et al. 2023) approaches. This framework would allow us to explore the broader impact of cyclic hypoxia on tumour development, not only in regulating tumour growth but also in influencing tumour invasion and metastasis (Saxena and Jolly 2019).

The results from our in silico serial passage assays highlight the role of damage repair capacity in shaping cancer cell responses and adaptation in fluctuating oxygen environments. Here, we have modelled pre-existing alteration of the damage repair capacity of cells, neglecting behavioural changes that may occur over the time scale of the experiments. In practice, cell cycle progression and damage repair are regulated at the genetic and epigenetic levels. While genetic mutations lead to irreversible changes in cancer cell behaviour, phenotypic changes are reversible and dynamically regulated, allowing cells to cope with unfavourable dynamic environments. For example, asymmetric damage segregation has been identified as a driver of cell-to-cell heterogeneity and a strategy to increase population-level fitness and counteract ageing under stress in bacteria, yeasts and stem cells (Vedel et al. 2016). In the context of cancer, there is evidence that replication stress leads to increased nonrandom segregation of damaged chromosomes (Xing et al. 2020) thus promoting genomic instability. It would be interesting to extend our modelling framework to include the finding of Xing et al. (2020) and study how different forms of hypoxia affect asymmetric cell division via replication stress, thus contributing to intratumour phenotypic heterogeneity (Buss et al. 2024; Jain et al. 2022). Other key players in cell adaptation to hypoxia are the hypoxia-inducible factors (HIFs). HIF-signalling has been linked to dedifferentiation and metabolic reprogramming of cancer cells (Saxena and Jolly 2019), which reduces damage accumulation while enhancing repair under hypoxia. Building on previous work on structured-population modelling (Ardaševa et al. 2020; Celora et al. 2023; Lorenzi and Painter 2022), it would be interesting to investigate the interplay between damage repair, phenotypic heterogeneity and cyclic hypoxia in shaping intratumour heterogeneity.

## Figures and Tables

**Algorithm 1:**
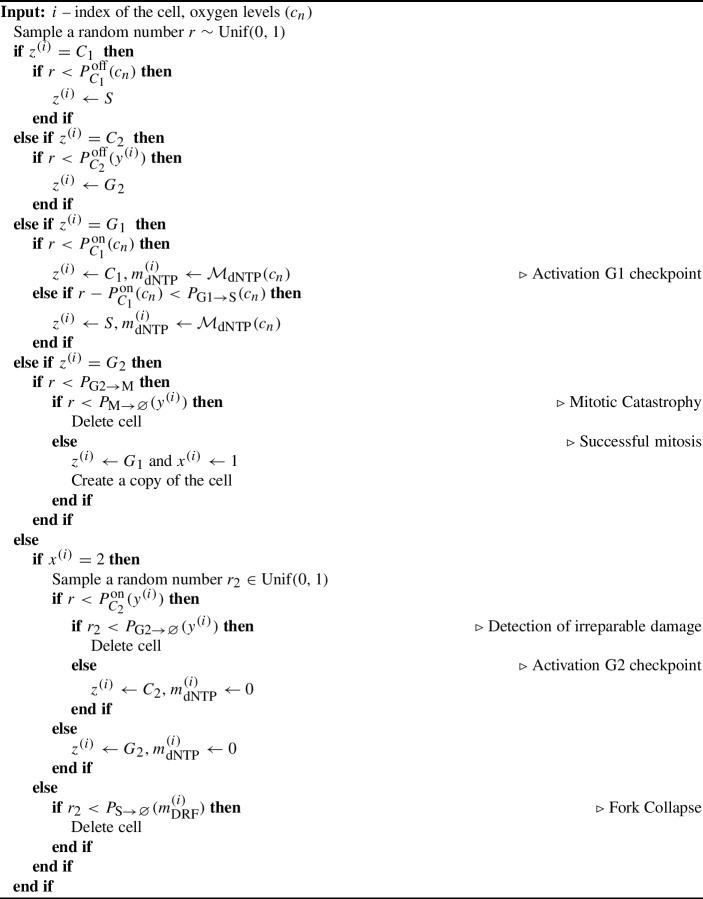
Pseudocode outlining the procedure used to simulate cell proliferation and death. The functions $P_{C_1}^{\text {on}/\text {off}}$, $P_{C_2}^{\text {on}/\text {off}}$, $P_{\text {G1}\rightarrow \text {S}}$, $P_{\text {G2}\rightarrow \text {M}}$, $P_{\text {M}\rightarrow \varnothing }$, $P_{\text {S}\rightarrow \varnothing }$ and $P_{\text {G2}\rightarrow \varnothing }$ are defined by Eqs. (8)-(9) and ${\mathcal {M}}_\text {dNTP}$ by Eq. (10c).

**Algorithm 2:**
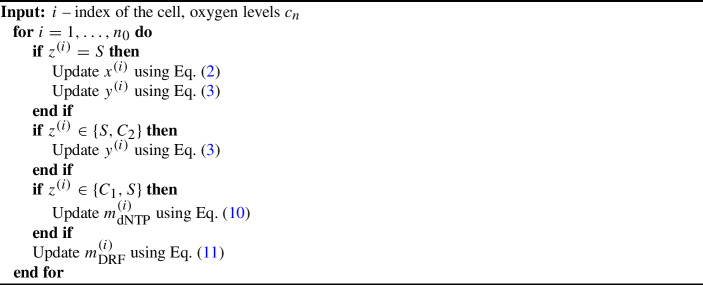
Pseudocode outlining the procedure used to simulate intracellular processes.

**Fig. 12:**
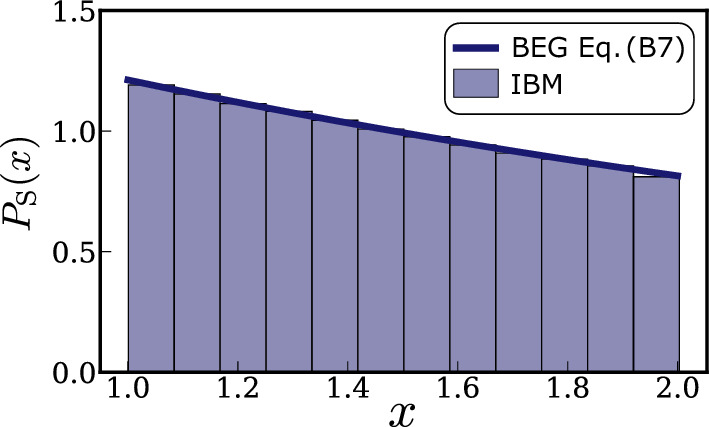
*S*-type cell DNA distribution $P_{\text {S}}(x)$ during balance exponential growth. The blue curve represents the theoretical prediction from Celora et al. (2022) (see Eq. (13)). The histogram is obtained from a simulation of the IB model (as in Fig. 7). While we simulate the model for 100 hours, we neglect the first 75 hours of the simulation when estimating the DNA distribution; this limits the influence of the initial conditions on the estimated distribution

**Algorithm 3:**
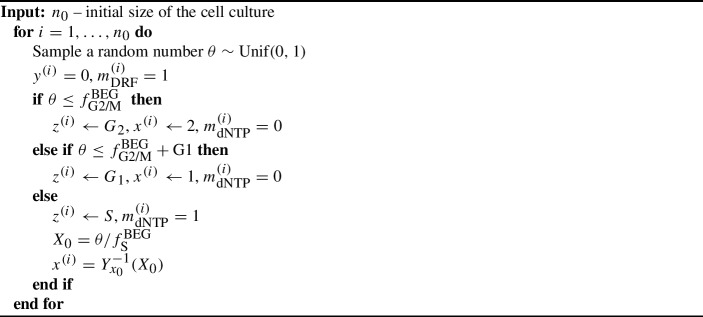
Pseudocode outlining the procedure used to initialise the simulations of our IB model. The function $Y^{-1}_{x_0}$ indicates the inverse of the cumulative DNA distribution $Y_{x_0}$ (see Eq. (14)).

**Fig. 13:**
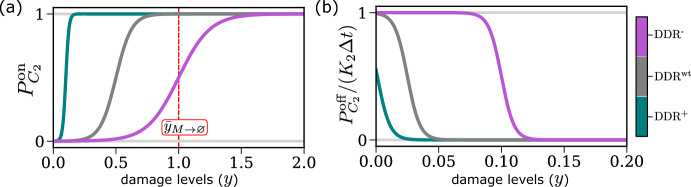
Plots of the profiles of **a** $P_{C_2}^\text {on}$ (Eq. (8e)) and **b** $P_{C_2}^{\text {off}}$ (Eq. (8d)) for cells with different damage repair capacity. The red dotted line in Fig. 13a indicates the curve $y={\bar{y}}_{M\rightarrow \varnothing }$, where ${\bar{y}}_{M\rightarrow \varnothing }$ corresponds to the threshold damage level for mitotic catastrophy. Parameter values are as indicated in Table 3 (Color figure online)

**Fig. 14:**
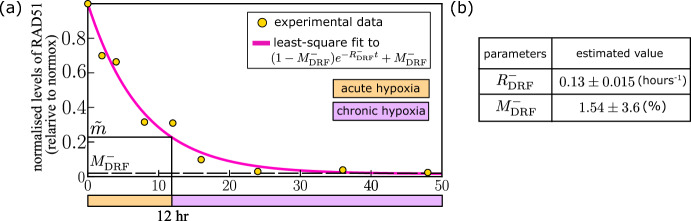
**a** Data from Pires et al. (2010a) on the time-evolution of expression levels of the DNA repair protein RAD51 in constant hypoxia (yellow dots). The pink curve indicates the value of the function $m_{\text {DRF} }(t)=(1-M_\text {DRF} ^-)e^{R_\text {DRF} ^-t}+M_\text {DRF} ^-$ for parameter values as specified in the panel (**b**). The functional form for $m_\text {DRF}$ is obtained by solving the deterministic version of Eq. (11) starting with $m_\text {DRF} (0)=1$ and setting $c(t)\equiv c_-<c_H$ for all $t>0$. **b** Estimates of the parameters $M_\text {DRF} ^-$ and $R_\text {DRF} ^-$ are obtained by fitting the function $m_{\text {DRF} }(t)=(1-M_\text {DRF} ^-)e^{R_\text {DRF} ^-t}+M_\text {DRF} ^-$ to the experimental data using the curve_fit function in the *SciPy* library in Python. For each parameter, we indicate the computed 67% confidence interval (Color figure online)

**Fig. 15:**
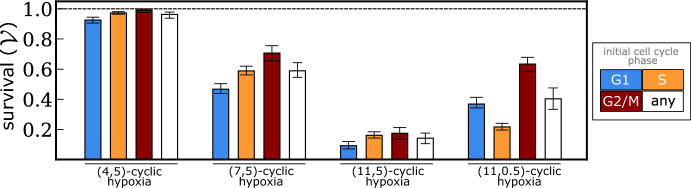
Characterising the dependence on survival of the initial cell cycle distribution. Barplots indicate the probability of survival, ${\mathcal {V}}$, estimated via simulation of clonogenic assay experiments (see Sect. 3.4.2) stratified by the cell cycle phase of the progenitor cell. We compare the results for four cyclic hypoxia conditions: (4,5), (7,5), (11,5), and (11,0.5)-cyclic hypoxia. Values of the parameters are as in Fig. 7

**Fig. 16:**
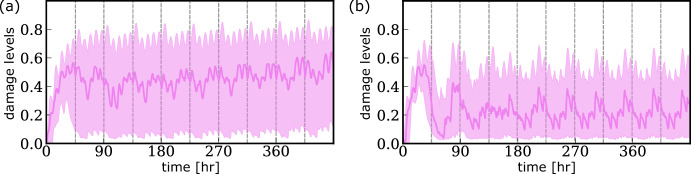
Plots of the time-evolution of the damage distribution during simulations of serial passage experiments in (4,5)-cyclic hypoxia for **a** co-culture and **b** control conditions. We indicate the median (see violet curve) and the interquartile range (see shaded area) of the damage distribution extracted from the simulations from Fig. 11. The vertical dotted lines indicate the time at which cells are passed (i.e., replated)

## Data Availability

In compliance with EPSRC’s open access initiative, the research materials supporting this publication can be accessed by contacting g.celora@ucl.ac.uk.

## A Detailed Implementation of in vitro Cancer Cell Dynamics in Hypoxia

We detail the implementation of cell proliferation and death, and intracellular processes in our individual-based (IB) model in Algorithm 1 and Algorithm 2. As shown in Fig. 3, for each cell, we first update its cell cycle state (as discussed in Appendix A.1). We then check for cell division/death (as discussed in Appendix A.2) and finally update its internal variables (as discussed in Appendix A.3). Given a sufficiently small time step $\Delta t$ and denoting by $c_n$ the oxygen levels at time $t_n$, the following rules are used to simulate cell cycle progression and cell fate decisions (i.e., cell death) in the time interval $\left[ t_{n},t_n+\Delta t\right)$:

### A.1 Cell-Cycle Transitions and Checkpoint Dynamics

At each time step, any surviving cell in $G_1$, $C_1$, *S*, or $C_2$ can transition to the next cell cycle phase, arrest due to activation of a checkpoint, or re-enter the cell cycle upon checkpoint deactivation. The dynamics of cells in states $G_1$ and $C_1$ are modelled as in Celora et al. (2022). New rules are introduced to describe the evolution of *S*, $G_2$ and $C_2$ cells (see Appendix A). Based on experimental evidence (see Sect. 2), cell cycle progression is implemented as follows:

- A cell in state $z^{(i)}=C_1$ may re-enter the cell cycle by transitioning to the S phase with probability
  8a $$\begin{aligned} P_{C_1}^{\text {off}}(c_n)=K_1\Delta t \ \sigma _+\left( c_n;c_H,s_{C_1}\right) , \end{aligned}$$
  where $\sigma _+$ is given by Eq. (1), $c_H$ is the oxygen threshold for hypoxia, and the positive constant $K_1$ denotes the maximum rate at which cells exit the $C_1$ state and initiate DNA synthesis by entering the *S* state. Eq. (8a) captures the inhibitory effect of hypoxia ($c<c_H$) on the $C_1\rightarrow S$ transition; based on previous work (Celora et al. 2022), we assume a switch-like behaviour and fix $0<s_{C_1}\ll 1$.
- A cell in state $z^{(i)}=G_1$ may arrest in the G1 phase ($z^{(i)} \rightarrow C_1$) or proceed to the S phase ($z^{(i)} \rightarrow S$) with probabilities
  8b $$\begin{aligned} P_{C_1}^{\text {on}}(c_n)&=k_1\Delta t \ \sigma _-\left( c_n;c_H,s_{C_1}\right) , \end{aligned}$$
  8c $$\begin{aligned} \quad P_{\text {G1}\rightarrow \text {S}}(c_n)&=k_1\Delta t-P_{C_1}^\text {on}(c_n). \end{aligned}$$
  In Eqs. (8b)-(8c), the positive constant $k_1$ represents the rate at which cells exit the G1 phase in oxygen-rich conditions, while $c_H$ and $s_{C_1}$ are as above. In Eq. (8b), the hypoxia-mediated activation of the G1 checkpoint is captured by the $\sigma _-$ activation function, which is defined as in Eq. (1).
- A cell in state $z^{(i)}=C_2$ may exit G2 arrest ($z^{(i)} \rightarrow G_2$) with probability which depends on its damage level $y^{(i)}$
  8d $$\begin{aligned} P_{C_2}^{\text {off}}(y^{(i)}) = K_2 \Delta t \ \sigma _-\left( y^{(i)};{\bar{y}}_{C_2}^{\text {off}}, s_{C_2}^\text {off}\right) \end{aligned}$$
  where the positive constants $K_2$, ${\bar{y}}^\text {off}_{C_2}$ and $s_{C_2}^\text {off}$ represent, respectively, the maximum rate at which cells leave the G2 checkpoint, the threshold damage level for G2 checkpoint deactivation and the sensitivity of the G2 checkpoint deactivation to damage levels. Eq. (8d) implies that cells are allowed to re-enter the cell cycle by transitioning to state $G_2$ only if their damage levels are sufficiently low. By setting ${\bar{y}}_{M\rightarrow \varnothing }\gg {\bar{y}}_{C_2}^{\text {off}}$, we ensure that cells exiting the G2 checkpoint will successfully undergo mitosis.
- A cell in state $z^{(i)}=S$, upon completing DNA synthesis ($x^{(i)}=2$), may arrest due to damage-mediated activation of the G2 checkpoint ($z^{(i)}\rightarrow C_2)$ or transition to the next cell cycle phase ($z^{(i)}\rightarrow G_2)$ with probabilities
  8e $$\begin{aligned} P_{C_2}^\text {on}(y^{(i)})&=\sigma _+\left( y^{(i)};{\bar{y}}_{C_2}^\text {on},s_{C_2}^\text {on}\right) , \end{aligned}$$
  8f $$\begin{aligned} P_{\text {S}\rightarrow \text {G2/M}}(y^{(i)})&=1-P_{C_2}^{\text {on}}(y^{(i)}), \end{aligned}$$
  where the positive constants ${\bar{y}}_{C_2}^\text {on}$ and $s_{C_2}^\text {on}$ represent, respectively, the threshold damage level for activation of the G2 checkpoint and the sensitivity of G2 checkpoint activation to damage levels. Eq. (8e) captures the graded damage-mediated activation of the G2 checkpoint (*i.e.*, transition to state $C_2$) that slows a cell’s progression through the G2 phase, to allow damage repair.

### A.2 Cell Fate Decision: Cell Death & Division

Based on experimental evidence (see Sect. 2), we include different forms of replication/cell death which are cell cycle specific. We assume that cells in the G1 phase (i.e., $z=G_1,C_1$) are not sensitive to hypoxia-mediated death, whereas cells in other cell-cycle states, $z^{(i)}\in \left\{ S,G_2,C_2\right\}$, die with probabilities $P_{z^{(i)}\rightarrow \varnothing }$ which depend on their damage level $y^{(i)}$ and/or their DRF expression levels $m_\text {DRF} ^{(i)}$ (see Fig. 3) in the following way.

- A cell that remains in the S phase may die due to fork collapse with probability
  9a $$\begin{aligned} P_{\text {S}\rightarrow \varnothing }(m_\text {DRF} ^{(i)})= \mu _\text {S}\Delta t\ \sigma _-\left( m^{(i)}_\text {DRF} ;{\bar{m}}_{\text {S}\rightarrow \varnothing },s_{\text {S}\rightarrow \varnothing }\right) , \end{aligned}$$
  where the positive constants $\mu _\text {S}$, ${\bar{m}}_{\text {S}\rightarrow \varnothing }$ and $s_{\text {S}\rightarrow \varnothing }$ represent, respectively, the maximum rate of cell death due to fork collapse, the threshold of DRF levels for activation of fork collapse, and the sensitivity of fork collapse to DRF levels. In line with the discussion in Section 2, fork collapse is regulated by the intracellular levels of DNA repair factors.
- A cell in state $z^{(i)}=G_2$ may attempt mitosis (*i.e.*, cell division) with probability
  9b $$\begin{aligned} P_{\text {G2}\rightarrow \text {M}}= k_2\Delta t, \end{aligned}$$
  where $k_2>0$ is the constant rate at which cells in state $G_2$ attempt mitosis. During this process, a cell may die via mitotic catastrophe (Matthews et al. 2022) due to the accumulated damage with probability
  9c $$\begin{aligned} P_{\text {M}\rightarrow \varnothing }(y^{(i)})&=\sigma _+\left( y^{(i)};{\bar{y}}_{\text {M}\rightarrow \varnothing },s_{\text {M}\rightarrow \varnothing }\right) . \end{aligned}$$
  Here, ${\bar{y}}_{M\rightarrow \varnothing }$ and $s_{M\rightarrow \varnothing }$ are positive constants representing, respectively, the threshold damage level for mitotic catastrophe and the sensitivity of mitotic catastrophe to damage levels. Without loss of generality, the damage levels *y* are rescaled so that ${\bar{y}}_{M\rightarrow \varnothing }=1$.
- A cell that enters the G2 checkpoint, *i.e.*, it has transitioned to state $C_2$, may permanently exit the cell-cycle due to accumulation of irreparable damage with probability
  9d $$\begin{aligned} P_{\text {G2}\rightarrow \varnothing }(y^{(i)})=\sigma _+\left( y^{(i)};{\bar{y}}_{\text {G2}\rightarrow \varnothing },s_{\text {G2}\rightarrow \varnothing }\right) . \end{aligned}$$
  In Eq. (9d), ${\bar{y}}_{\text {G2}\rightarrow \varnothing }$ and $s_{\text {G2}\rightarrow \varnothing }$ are positive constants representing respectively the threshold damage level for replicative death upon activation of the G2 checkpoint and the sensitivity of replicative death to damage levels. We here take ${\bar{y}}_{\text {G2}\rightarrow \varnothing }>{\bar{y}}_{\text {M}\rightarrow \varnothing }$ to account for the protective effect of the G2 checkpoint activation. More specifically, for a given value of $y^{(i)}$, a cell’s chances of successfully dividing are increased by activation of the G2 checkpoint. Given the form of Eqs. (9c)-(9d), the benefit of activation of the G2 checkpoint is maximal for intermediate values of cell damage, and minimal for very low ($y\ll {\bar{y}}_{\text {M}\rightarrow \varnothing }$) and very high damage ($y\gg {\bar{y}}_{\text {G2}\rightarrow \varnothing }$) levels.

When a cell dies, it is simply removed from the system. When a cell divides, the original parent cell is removed and two $G_1$ daughter cells are added. These inherit the values of the state variables *y*, $m_\text {dNTP}$ and $m_\text {DRF}$ from the parent cell.

### A.3 Modelling the Impact of Hypoxia on Intracellular Factors

At each time step, the dynamics of intracellular processes (namely, DNA synthesis, damage repair, dNTP and DRF synthesis/degradation) are simulated within each cell following the procedure detailed in Algorithm 2. Details on the modelling of DNA synthesis and damage repair have been discussed in Sections  3.3.1 and 3.3.2, respectively. We now explain how we account for the impact of hypoxia on dNTP levels ($m_\text {dNTP}$) and DRF levels ($m_\text {DRF}$) in our IB model.

#### A.3.1 Modelling the Dynamics of Intracellular dNTP Levels

Levels of intracellular dNTPs are known to increase upon entry to the S phase (before initiation of DNA synthesis) and to decrease when a cell completes DNA synthesis (Stillman 2013). In line with these observations, we fix $m_{\text {dNTP} }^{(i)}=0$ for all cells *i* except those in states $C_1$ and *S*. Since de-novo production of dNTPs is impaired under low-oxygen, we assume that the change in $m^{(i)}_\text {dNTP}$ over a time-step $\Delta t$ satisfies:

10a $$\begin{aligned} m^{(i)}_\text {dNTP} (t_{n+1})=m^{(i)}_\text {dNTP} (t_n)+(1+\Theta )\Delta m^{(i)}_\text {dNTP} , \end{aligned}$$

where the multiplicative noise term $\Theta \sim {\mathcal {N}}(0,\sigma )$ accounts for intercellular variability and we define

10b $$\begin{aligned} \Delta m^{(i)}_{\text {dNTP} }&={\left\{ \begin{array}{ll} {\mathcal {R}}_{\text {dNTP} }(c_n) \Delta t\ [{\mathcal {M}}_{\text {dNTP} }(c_n)- m^{(i)}_{\text {dNTP} }(t_n)], & \quad z^{(i)}=C_1,S,\\ 0,& \quad \text{ otherwise }. \end{array}\right. } \end{aligned}$$

In Eq. (10b), the function ${\mathcal {M}}_\text {dNTP} (c)\le 1$ indicates the baseline expression levels of dNTPs as a function of oxygen, while the positive function ${\mathcal {R}}_\text {dNTP} (c)$ indicates the rate at which dNTP levels relax to such baseline values as a function of oxygen. Following Celora et al. (2022), we account for differences in the dynamics of dNTP levels under physiological and hypoxic oxygen conditions by setting:

10c $$\begin{aligned} {\mathcal {R}}_\text {dNTP} (c)={\left\{ \begin{array}{ll} R_\text {dNTP} ^+,\quad c>c_H,\\ R_\text {dNTP} ^-,\quad c<c_H, \end{array}\right. },\quad {\mathcal {M}}_\text {dNTP} (c)={\left\{ \begin{array}{ll} 1,\quad c>c_H,\\ M^-_\text {dNTP} ,\quad c<c_H, \end{array}\right. }. \end{aligned}$$

In Eq. (10c) the positive constants $R_\text {dNTP} ^+$, $R_\text {dNTP} ^-$ and $M_\text {dNTP} ^{-}<1$ represent, respectively, the rate of recovery under oxygen-rich conditions, the rate of inhibition in hypoxia, and baseline expression levels under hypoxia. When a cell exits the $G_1$ state, its dNTP levels are set to the baseline value ${\mathcal {M}}_\text {dNTP} (c)$.

#### A.3.2 Modelling the Dynamics of Intracellular DRF Levels

We assume that the dynamics of the DNA repair factors do not depend on the cell cycle state (Bindra et al. 2004) and are regulated uniquely by oxygen levels (see Sect. 2). As for $m_\text {dNTP}$, we model the impact of hypoxia on the expression level $m^{(i)}_{\text {DRF} }$ in cell *i* (see Fig. 1) by using the following rule to update the DRF expression levels in cell *i* from time $t_n$ to time $t_{n+1}$:

11a $$\begin{aligned} m^{(i)}_\text {DRF} (t_{n+1})=m^{(i)}_\text {DRF} (t_n)+(1+\Theta )\Delta m^{(i)}_\text {DRF} , \end{aligned}$$

where $$\Theta \sim {\mathcal {N}}(0,\sigma )$$,

11b $$\begin{aligned} \Delta m^{(i)}_{\text {DRF} }&={\mathcal {R}}_\text {DRF} (c_n)\Delta t\ {[}{\mathcal {M}}_\text {DRF} (c_n)-m^{(i)}_\text {DRF} (t_n)], \end{aligned}$$

11c $$\begin{aligned} {\mathcal {R}}_\text {DRF} (c)&={\left\{ \begin{array}{ll} R_\text {DRF} ^{+},\quad c>c_H,\\ R_\text {DRF} ^{-},\quad c<c_H, \end{array}\right. },\quad {\mathcal {M}}_\text {DRF} (c)={\left\{ \begin{array}{ll} 1,\quad c>c_H,\\ M^-_\text {DRF} ,\quad c<c_H, \end{array}\right. }, \end{aligned}$$

and $R_\text {DRF} ^{+}$, $R_\text {DRF} ^{-}$ and $M_\text {DRF} ^{-}<1$ are constant positive parameters representing, respectively, the rate at which DRF levels increase under oxygen-rich conditions, the rate at which DRF levels decrease under hypoxia and the baseline expression levels of DRF under hypoxia. As above, we account for intercellular variability by adding multiplicative noise in Eq. (11a).

## B Balanced exponential growth (BEG)

The concept of balanced (or asynchronous) exponential growth (BEG) was first introduced by cell biologists to describe the growth of cell populations (Webb 1987). This regime describes a situation where the total number of cells, *N*, grows exponentially at a constant rate $\lambda ^{\text {BEG}}$ (i.e., $N\propto \exp \left[ \lambda ^{\text {BEG}} t\right]$) and the distribution of cells in the different phases of the cell-cycle tends to a constant profile, which is independent of how the cells were initially distributed along the cell cycle. Experimentally, BEG is observed in cultures of cells and bacteria at low density (i.e., in the absence of competition for space and nutrients).

In an oxygen-rich environment (i.e., in physiological conditions), cells have no damage (see Fig. 6a), and levels of dNTP and DRF are sufficiently high to guarantee normal cell cycle progress. As a result, cell death is negligible and the number of cells in the checkpoint compartments will tend to zero (i.e., no cells in states $C_1$ and $C_2$). The only difference in the intracellular state of cells is, therefore, their DNA content, *x*. We investigated BEG for DNA-structured populations in our previous work (Celora et al. 2022) and found that the cell cycle distribution (i.e., the fraction of cells in each cell cycle phase: $f^\text {BEG}_{\text {G1}}$, $f^\text {BEG}_\text {S}$ and $f^\text {BEG}_{\text {G2/M}}$) and the growth rate during BEG are related to model parameters as follows

12a $$\begin{aligned} f^{\text {BEG}}_{\text {G2/M}}&=\frac{\lambda ^{\text {BEG}}}{k_2}, \end{aligned}$$

12b $$\begin{aligned} f^{\text {BEG}}_{\text {G1}}&=\frac{2\lambda ^{\text {BEG}}}{\lambda ^{\text {BEG}}+k_1}, \end{aligned}$$

12c $$\begin{aligned} f^{\text {BEG}}_{\text {S}}&=1-f^{\text {BEG}}_{\text {G2/M}}-f^{\text {BEG}}_{\text {G1}}, \end{aligned}$$

12d $$\begin{aligned} (\lambda ^{\text {BEG}}+k_1)(\lambda ^{\text {BEG}}+k_2)&-2k_1k_2e^{-\lambda ^{\text {BEG}}/{\bar{v}}_x} = 0. \end{aligned}$$

In Eq. (12), the positive constants $k_1$, $k_2$ and ${\bar{v}}_x$ are as in Tables 3-4. The existence and uniqueness of real solutions for Eq. (12d) are discussed in Celora et al. (2022). As shown in Figure 7a, the asymptotic behaviour predicted by the IB model agrees with Eq. (12). Using the results in Celora et al. (2022), we can also derive the DNA distribution of cells in the S phase

13 $$\begin{aligned} P_{\text {S}}(x) = k_s\frac{e^{-k_s(x-1)}}{1-e^{-k_s}}, \quad \text{ where } k_s=\frac{\lambda ^{\text {BEG}}}{{\bar{v}}_x}. \end{aligned}$$

Here $P_{\text {S}}(x)$ is the probability that a randomly sampled *S*-type cell has DNA content $x^{(i)}=x$. In Eq. (13), the variable *x* has a truncated exponential distribution on the interval [1, 2] with rate $k_s$, $x\sim \text {Exp}_{[1,2]}(k_s)$. In Fig. 12, we compare Eq. (13) with the shape of $P_{\text {S}}$ estimated from simulations of the IBM model for physiological oxygen levels. The two profiles are in excellent agreement.

We start each simulation with 100 cells. At the beginning of each simulation, an initial state is assigned to each cell as outlined in Algorithm 3. The cell cycle state $z^{(i)}$ and DNA content $x^{(i)}$ are assigned by sampling from the BEG distribution. This requires the values of $f^\text {BEG}_{G1,S,G2/M}$ and $k_s$ (see Table 6) and the definition of the cumulative DNA distribution $Y_{x_0}$

14 $$\begin{aligned} Y_{x_0}=\int _1^{x_0} P_{\text {S}}(\xi )d\xi = \frac{1-e^{-k_s(x-x_0)}}{1-e^{-k_s}}, \quad x_0 \in [1,2] \end{aligned}$$

where $P_\text {S}$ is defined by Eq. (13). The other state variables are initialised to their default physiological values, so that $y^{(i)}=0$, $m^{(i)}_{\text {DRF} }=1$, and $m^{(i)}_\text {dNTP} =1$ if $z^{(i)}=S$ and $m^{(i)}_\text {dNTP} =0$ otherwise.

### B.1 Balanced Exponential Growth in Periodic Environment

If we consider an asynchronous population of cells that grows in a periodically changing environment in the absence of competition then, the total number of cells can be described by the following ordinary differential equations:

15 $$\begin{aligned} \frac{dN}{dt}= r(t) N(t), \end{aligned}$$

where *r*(*t*) is a ${\mathcal {T}}$-periodic function and represents the instantaneous growth rate. As the instantaneous growth rate *r*(*t*) captures information about fluctuations in the population size, it fails to characterise the long-term dynamics of the population (i.e., whether it will grow or go extinct). A more natural and informative way of studying the evolution of *N* in periodically changing conditions is to look at the overall change in the population over a period ${\mathcal {T}}$. Solving Eq. (15) in the time interval $[t,t+{\mathcal {T}})$ we find that:

16 $$\begin{aligned} N(t+{\mathcal {T}})=e^{R(t)} N(t), \quad R(t)=\int _{t}^{t+{\mathcal {T}}} r(\xi ) d\xi . \end{aligned}$$

Since *r*(*t*) is a periodic function, it follows that *R*(*t*) is constant since $R'(t)=r(t+{\mathcal {T}})-r(t)=0$. Hence, we can define the constant population growth rate $\lambda =R/{\mathcal {T}}$. Then we can rewrite Eq. (16) as:

17 $$\begin{aligned} N(t+{\mathcal {T}}) = e^{\lambda (n_{\mathcal {T}}+1) {\mathcal {T}}}N(t_0),\quad n_{\mathcal {T}}=\left\lfloor \frac{t}{{\mathcal {T}}}\right\rfloor , \quad t_0=t-n_{\mathcal {T}}{\mathcal {T}}, \end{aligned}$$

where $\lfloor \cdot \rfloor$ is the floor function. Inspecting Eq. (17), it is now apparent that for $\lambda <0$, the population is driven to extinction, while if $\lambda >0$ it grows unbounded. The time-averaged growth rate $\lambda$ is therefore able to capture the long-term dynamics of the population.

## C Parameter values

Tables 3-6 contain the model parameters used in the simulations. Where multiple values are indicated, these correspond to cell populations with different damage repair capacities (see Sect. 3.4.1 and Sect. 4.3). Where no reference is given, the parameter values have been chosen to produce biologically reasonable behaviour and a justification is given. In particular, the values were chosen to give growth and cell-cycle dynamics in line with experimental and theoretical predictions  (Celora et al. 2022; Bader et al. 2021b).

### C.1 Cell Cycle Transitions, Checkpoint Dynamics and Cell Fate Decisions

Table 3 lists the values of model parameters associated with cell cycle transition and activation/deactivation of cell cycle checkpoints, i.e., Eqs. (8), and cell-fate decisions, i.e., Eqs. (9).

**Table 3:** Summary of the parameters associated with cell cycle progression, checkpoint dynamics and cell-fate decisions and the values used in the simulations (see Eqs. (8)-(9))

	Description	Typical value(s)	Justification (Refs)
$\Delta t$	Timestep	1/50 (hours)	sufficiently small to capture the time-scale of all processes
$k_1$	Rate at which cells exit $G_1$	0.195 ($\hbox {hours}^{-1}$)	Celora et al. (2022)
$K_1$	Maximum rate at which cells leave the G1 checkpoint	1.00 ($\hbox {hours}^{-1}$)	Celora et al. (2022)
$c_H$	Hypoxia threshold	$\approx 1.0$ (% $\hbox {O}_{2}$)	Celora et al. (2022)
$s_{C_1}$	Sensitivity of G1 checkpoint to oxygen levels	0.01 (% $\hbox {O}_{2}$)	Taken to be smaller than $c_H$
$s_{C_2}^{\text {off}}$	Sensitivity deactivation of G2 checkpoint to damage levels	0.01 (a.u.)	Taken to be smaller than ${\bar{y}}_{C_2}^{\text {off}}$
$K_2$	Maximum rate at which cells leave the G2 checkpoint	1.00 ($\hbox {hours}^{-1}$)	Taken to be the same as $K_1$
${\bar{y}}_{C_2}^{\text {on}}$	Threshold damage level for activation G2 checkpoint	$\hbox {DDR}^{-}$ $\rightarrow$ 1.0 (a.u.)	(see Sect. 3.4.1)
		$\hbox {DDR}^{\textrm{wt}}$ $\rightarrow$ 0.5 (a.u.)	
		$\hbox {DDR}^{+}$ $\rightarrow$ 0.1 (a.u.)	
$s_{C_2}^{\text {on}}$	Sensitivity of G2 checkpoint activation to cellular damage levels	$\hbox {DDR}^{-}$ $\rightarrow$ 0.250 (a.u.)	Taken to be ${\bar{y}}_{C_2}^{\text {on}}/4$
		$\hbox {DDR}^{\textrm{wt}}$ $\rightarrow$ 0.125 (a.u.)	
		$\hbox {DDR}^{+}$ $\rightarrow$ 0.025 (a.u.)	
${\bar{y}}_{C_2}^{\text {off}}$	Threshold damage level for deactivation of G2 checkpoint	$\hbox {DDR}^{-}$ $\rightarrow$ 0.100 (a.u.)	Taken to be smaller than ${\bar{y}}_{C_2}^{\text {on}}$
		$\hbox {DDR}^{\textrm{wt}}$ $\rightarrow$ 0.025 (a.u.)	
		$\hbox {DDR}^{+}$ $\rightarrow$ 0.005 (a.u.)	
$k_2$	Rate at which $G_2$-cells attempt mitosis	0.22 ($\hbox {hours}^{-1}$)	Celora et al. (2022)
$\mu _s$	Maximum rate of cell death due to fork collapse in the S phase	0.05 ($\hbox {hours}^{-1}$)	match % of apoptotic cells after 20 hr exposure to hypoxia (Bader et al. 2021b)
${\bar{m}}_{S\rightarrow \varnothing }$	Threshold level of DRFs for cell death in the S phase	0.22 (a.u.)	corresponds to $m_\text {DRF}$ levels after 12 hr in hypoxia (see Fig. 14)
${\bar{y}}_{M\rightarrow \varnothing }$	Threshold damage level for mitotic catastrophe	1.00 (a.u.)	normalised
${\bar{y}}_{\text {G2}\rightarrow \varnothing }$	Threshold level damage level for cell death upon G2 checkpoint activation	2.50 (a.u.)	taken to be greater than ${\bar{y}}_{M\rightarrow \varnothing }$
$\epsilon _{\text {S}\rightarrow \varnothing }$	Sensitivity of *S*-cell death to changes in DRF levels	0.05 (a.u.)	taken to be less than half of ${\bar{m}}_{\text {S}\rightarrow \varnothing }$
$\epsilon _{\text {M}\rightarrow \varnothing }$	Sensitivity of mitotic catastrophe to damage levels	0.25 (a.u)	taken to be less than half of ${\bar{y}}_{\text {M}\rightarrow \varnothing }$
$\epsilon _{\text {G2}\rightarrow \varnothing }$	Sensitivity of cell-death upon G2 checkpoint activation to damage levels	1.00 (a.u)	taken to be less than half of ${\bar{y}}_{\text {G2}\rightarrow \varnothing }$

Typical values are given for the RKO cancer cell line. Multiple values are listed for those parameters that we change to model cancer cells with different damage repair capacities (see Fig. 13). The abbreviation (a.u.) stands for arbitrary units

As mentioned in Sect. 2, heterogeneity in the regulation of the DNA damage response (DDR) is commonly found in in vivo tumours. We model alteration to DDR response by changing model parameters associated with the probability of activation ($P_{C_2}^{\text {on}}$) and deactivation ($P_{C_2}^{\text {off}}$) of the G2 checkpoint in response to damage (see Eqs. (8d)-(8e)). To model enhanced DDR activation, we decrease ${\bar{y}}_{C_2}^\text {on}$, $s_{C_2}^\text {on}$ and ${\bar{y}}_{C_2}^\text {off}$ with respect to their default values. To model silencing of DDR signalling, we instead increase ${\bar{y}}_{C_2}^\text {on}$, $s_{C_2}^\text {on}$ and ${\bar{y}}_{C_2}^\text {off}$ with respect to their default values. Fig. 13 shows the profile of $P_{C_2}^{\text {on}}$ and $P_{C_2}^{\text {off}}$ in the three cases: default ($\hbox {DDR}^{\textrm{wt}}$ cells), enhanced DDR activation ($\hbox {DDR}^{+}$ cells) and silenced DDR signalling ($\hbox {DDR}^{-}$ cells).

### C.2 Intracellular Dynamics

Table 4 lists the values of model parameters associated with intracellular dynamics, i.e., Eqs. (2)-(3) and (10)-(11). We estimate the parameters associated with the expected evolution of repair protein expression levels, $m_\text {DRF} (t)$ (see Eq. (11)), using the data from Pires et al. (2010a) on the time-evolution of expression levels of the DNA repair protein RAD51 in RKO cells in constant hypoxia. While in our model $m_\text {DRF} (t)$ corresponds to the expression of multiple different damage repair proteins, we assume they behave similarly in response to hypoxia and use RAD51 dynamics as a prototypical response. As shown in Fig. 14, the expression levels relative to physiological conditions show a clear trend: the expression levels decrease monotonically as the period of exposure to hypoxia increases. The profile can be fitted to an exponential function (see the continuous curve in Fig. 14) justifying the functional form chosen for $m_\text {DRF} (t)$ (see Eq. (11b)). We estimate model parameters by fitting the exponential function ($m_{\text {DRF} }(t)=(1-M^-_{\text {DRF} })e^{-R^-_{\text {DRF} }t}+M^-_{\text {DRF} }$) to the experimental data. We could not find similarly detailed data for RKO cells to estimate $R^+_\text {DRF}$, i.e., the rate at which protein expression levels are restored upon reoxygenation. Estimates of $R^+_\text {DRF}$ were informed by Western-blot data from Bindra et al. (2004). We note that these experiments were not conducted on the RKO cell line.

**Table 4:** Summary of the parameters associated with the evolution of intracellular levels of dNTP and DRF used in the simulations that we can estimate from the literature

	Description	Typical value	Justification (Refs)
$\Delta t$	Timestep	1/50 (hours)	as in Table 3
$\gamma$	Rate at which DNA damage is accumulated in response to replication stress	0.2 ($\hbox {hours}^{-1}$)	match activation G2 checkpoint in 2/2 cyclic hypoxia Celora et al. (2022)
${\bar{v}}_{x}$	Maximum velocity of DNA synthesis	0.083 ($\hbox {hours}^{-1}$)	Celora et al. (2022)
${\bar{v}}_{y}$	Rate of damage repair in physiological levels	0.3 ($\hbox {hours}^{-1}$)	in line with values estimated in at low radiation Liu et al. (2021)*
$R_\text {DRF} ^{-}$	Rate of change in the expression of DRFs for $c<c_H$	0.13 ($\hbox {hours}^{-1}$)	Fig. 14 Pires et al. (2010a)
$R_\text {DRF} ^+$	Rate of change in the expression of DRFs for $c>c_H$	0.05 ($\hbox {hours}^{-1}$)	$\approx 90\%$ recovery in 48 hours (Bindra et al. 2004)*
$R_\text {dNTP} ^-$	Rate of change in the intracellular levels of dNTPs for $c<c_H$	0.3 ($\hbox {hours}^{-1}$)	Celora et al. (2022)
$R_\text {dNTP} ^+$	Rate of change in the intracellular levels of dNTPs for $c>c_H$	0.26 ($\hbox {hours}^{-1}$)	Celora et al. (2022)
$M_\text {DRF} ^{-}$	Equilibrium levels of DRFs expression levels in hypoxia	0.0154 (a.u.)	Fig. 14 Pires et al. (2010a)
$M_\text {dNTP} ^{-}$	equilibrium levels of dNTPs levels in hypoxia	0.06 (a.u.)	minimal rate of DNA synthesis as in Celora et al. (2022)

Typical values are given for the RKO cancer cell line except those taken from other cell lines (indicated with *)

Using the data in Fig. 14, we find that $m_{\text {DRF} }$ drops to ${\tilde{m}}=0.223$ after 12 hours in hypoxia. As discussed in Sect. 2, experimentally it is observed that, when exposed to hypoxia for more than 12 hours, cells become sensitive to the collapse of replication forks and repress repair mechanisms. Accordingly, we require that the death rate for cells in the S phase is half of its maximum value when cells are exposed to constant hypoxia for 12 hours, i.e., we set ${\bar{m}}_{S\rightarrow \varnothing }={\tilde{m}}$ in Eq. (9a).

### C.3 Oxygen Dynamics in the Chamber

Table 5 lists the values of model parameters used to simulate the evolution of oxygen levels within the oxygen chamber for different experimental setups (see Eqs. (4)).

**Table 5:** Summary of the parameters associated with the evolution of oxygen levels within the chamber used in the simulations (see Eqs. (4))

	Description	Typical value	Justification (Refs)
$c_H$	Hypoxia threshold	$\approx 1.0$ (% $\hbox {O}_{2}$)	Celora et al. (2022)
$c_+$	Re-oxygenation oxygen levels	2.0 (% $\hbox {O}_{2}$)	Celora et al. (2022)
$c_-$	Minimum oxygen levels	0.1 (% $\hbox {O}_{2}$)	Celora et al. (2022)
$\lambda _c$	Rate at which oxygen levels equilibrate	10 ($\hbox {hours}^{-1}$)	Equilibration takes $\approx$ 10 min

### C.4 Initial Conditions: BEG

Table 6 lists the values of model parameters used to initialise the numerical simulations (details can be found in Sect. 1).

**Table 6:** Summary of the parameters associated with the initialisation of the numerical simulations (see Sect. 1)

	Description	Typical value	Justification (Refs)
$f^+_{G1}$	Initial fraction of cells in the G1 phase	29 (%)	Celora et al. (2022)
$f^+_{S}$	Initial fraction of cells in the S phase	56 (%)	Celora et al. (2022)
$f^+_{G2}$	Initial fraction of cells in the G2 phase	15 (%)	Celora et al. (2022)
$k_s$	Rate at which cell leaves the S phase in the BEG regime	$\approx 0.4 (\textrm{hours}^{-1})$	Eq. (13) with ${\bar{v}}_x$ and $\lambda ^{\text {BEG}}$ from Celora et al. (2022)

## D Initial Cell Cycle State Influences Cell Survival

Figure 15 shows the estimates of survival obtained via numerical simulations of clonogenic assays (see Sect. 3.4.2) stratified by the cell cycle phase of the initially seeded cells. We compare results for four cyclic hypoxia conditions; namely, (4,5)-, (7,5)- , (11,5)- and (11.05)-cyclic hypoxia. For oxygen conditions where overall population survival is high (e.g., (4,5)-cyclic hypoxia) or low (e.g., (11,5)-cyclic hypoxia), there is no significant difference in cell survival for cells starting in different cell cycle phases. Larger deviations are observed for conditions where the overall population survival ${\mathcal {V}}\approx 0.5$ (e.g., (7,5)-cyclic hypoxia). In this example, progenitor cells initially in the late stages of the cell cycle – i.e. in the S and G2/M phases – are more likely to survive. As the duration of ${\mathcal {T}}_R$ decreases and we approach conditions analogous to constant hypoxia (e.g., (11,0.5)-cyclic hypoxia), we observe an even stronger correlation between cell survival and the initial cell cycle phase of the progenitor cell. Overall, the results presented in Fig. 15 highlight the importance of deconvoluting cell cycle-specific sensitivities when accessing survival in toxic environments.

## E Long-Term Damage Distribution in Serial Passage Experiments.

In Sect. 4.3 we claim that, during serial passage experiments in cyclic hypoxia, the distribution of damage within the population does not converge to a stationary distribution at long times; rather it fluctuates with the oxygen levels, and it eventually settles to a time-periodic function whose period coincides with the interval between passages of the cell population. In this section, we present additional results in support of our claim.

Figure 16 illustrates the time-evolution of the median and IQR of the damage distribution in the population during serial passage assays (see Sect. 3.4.3) for cells exposed to (4,5)-cyclic hypoxia. We report the simulated time-evolution for both the co-culturing (see Fig. 16a) and control experiments (see Fig. 16b). The results show that the mean and interquartile range of the damage distribution do not settle to stationary values but rather they fluctuate over time with the oxygen levels. At long times, the distribution converges to a time-periodic function, whose period is equivalent to the interval between contiguous passages of the cell population (here 45 hours).

## References

[CR1] Aguirre-Ghiso JA (2007) Models, mechanisms and clinical evidence for cancer dormancy. Nat Rev Cancer 7(11):834–846. 10.1038/nrc2256 17957189 10.1038/nrc2256 PMC2519109

[CR2] Ardaševa A, Gatenby RA, Anderson ARA et al (2020) A mathematical dissection of the adaptation of cell populations to fluctuating oxygen levels. Bull Math Biol 82(6):81. 10.1007/s11538-020-00754-7 32556703 10.1007/s11538-020-00754-7

[CR3] Bader SB, Dewhirst M, Hammond E (2021) Cyclic hypoxia: an update on its characteristics, methods to measure it and biological implications in cancer. Cancers 13(1):1–20. 10.3390/cancers13010023 10.3390/cancers13010023 PMC7793090 33374581

[CR4] Bader SB, Ma TS, Simpson CJ et al (2021) Replication catastrophe induced by cyclic hypoxia leads to increased APOBEC3B activity. Nucleic Acids Res 49(13):7492–7506. 10.1093/nar/gkab551 34197599 10.1093/nar/gkab551 PMC8287932

[CR5] Begg K, Tavassoli M (2020) Inside the hypoxic tumour: reprogramming of the DDR and radioresistance. Cell Death Discovery 6(1):1–15. 10.1038/s41420-020-00311-0 10.1038/s41420-020-00311-0 PMC7434912 32864165

[CR6] Ben-Ami Y, Atkinson GW, Pitt-Francis JM et al (2022) Structural features of microvascular networks trigger blood flow oscillations. Bull Math Biol 84(8):85. 10.1007/s11538-022-01046-y 35802265 10.1007/s11538-022-01046-y PMC9270315

[CR7] Bindra RS, Schaffer PJ, Meng A et al (2004) Down-regulation of Rad51 and decreased homologous recombination in hypoxic cancer cells. Mol Cell Biol 24(19):8504–8518. 10.1128/MCB.24.19.8504-8518.2004 15367671 10.1128/MCB.24.19.8504-8518.2004 PMC516750

[CR8] Bristow RG, Hill RP (2008) Hypoxia and metabolism: hypoxia, DNA repair and genetic instability. Nat Rev Cancer 8(3):180–192. 10.1038/nrc2344 18273037 10.1038/nrc2344

[CR9] Bull JA, Mech F, Quaiser T et al (2020) Mathematical modelling reveals cellular dynamics within tumour spheroids. PLoS Comput Biol 16(8):e1007961. 10.1371/journal.pcbi.1007961 32810174 10.1371/journal.pcbi.1007961 PMC7455028

[CR10] Buss JH, Begnini KR, Lenz G (2024) The contribution of asymmetric cell division to phenotypic heterogeneity in cancer. J Cell Sci 137(5):jcs261400. 10.1242/jcs.261400 38334041 10.1242/jcs.261400

[CR11] Celora GL (2022) Modelling the impact of cyclic hypoxia on cell-cycle regulation in cancer cells. PhD thesis, University of Oxford, URL https://ora.ox.ac.uk/objects/uuid:eff0168a-a623-43da-bc6f-373e540bd2c3

[CR12] Celora GL, Bader S, Hammond EM et al (2022) A DNA-structured mathematical model of cell-cycle progression in cyclic hypoxia. J Theor Biol 545:111104. 10.1016/J.JTBI.2022.111104 35337794 10.1016/j.jtbi.2022.111104

[CR13] Celora GL, Byrne HM, Kevrekidis PG (2023) Spatio-temporal modelling of phenotypic heterogeneity in tumour tissues and its impact on radiotherapy treatment. J Theor Biol 556:111248. 10.1016/j.jtbi.2022.111248 36150537 10.1016/j.jtbi.2022.111248

[CR14] Emami Nejad A, Najafgholian S, Rostami A et al (2021) The role of hypoxia in the tumor microenvironment and development of cancer stem cell: a novel approach to developing treatment. Cancer Cell Int 21(1):62. 10.1186/s12935-020-01719-5 33472628 10.1186/s12935-020-01719-5 PMC7816485

[CR15] Ferrell JE, Tsai TYC, Yang Q (2011) Modeling the cell cycle: Why do certain circuits oscillate? Cell 144(6):874–885. 10.1016/j.cell.2011.03.006 21414480 10.1016/j.cell.2011.03.006

[CR16] Foskolou IP, Jorgensen C, Leszczynska KB et al (2017) Ribonucleotide reductase requires subunit switching in hypoxia to maintain DNA replication. Mol Cell 66(2):206–220. 10.1016/j.molcel.2017.03.005 28416140 10.1016/j.molcel.2017.03.005 PMC5405111

[CR17] Franken NAP, Rodermond HM, Stap J et al (2006) Clonogenic assay of cells in vitro. Nat Protoc 1(5):2315–2319. 10.1038/nprot.2006.339 17406473 10.1038/nprot.2006.339

[CR18] Ghaffarizadeh A, Heiland R, Friedman SH et al (2018) PhysiCell: an open source physics-based cell simulator for 3-D multicellular systems. PLoS Comput Biol 14(2):e1005991. 10.1371/journal.pcbi.1005991 29474446 10.1371/journal.pcbi.1005991 PMC5841829

[CR19] Goto T, Kaida A, Miura M (2015) Visualizing cell-cycle kinetics after hypoxia/reoxygenation in HeLa cells expressing fluorescent ubiquitination-based cell cycle indicator (Fucci). Exp Cell Res 339(2):389–396. 10.1016/j.yexcr.2015.10.019 26500111 10.1016/j.yexcr.2015.10.019

[CR20] Hamis S, Yates J, Chaplain MAJ et al (2021) Targeting cellular DNA damage responses in cancer: an in vitro-calibrated agent-based model simulating monolayer and spheroid treatment responses to ATR-inhibiting drugs. Bull Math Biol 83(10):103. 10.1007/s11538-021-00935-y 34459993 10.1007/s11538-021-00935-y PMC8405495

[CR21] Hanahan D (2022) Hallmarks of cancer: new dimensions. Cancer Discov 12(1):31–46. 10.1158/2159-8290.CD-21-1059 35022204 10.1158/2159-8290.CD-21-1059

[CR22] Höckel M, Vaupel P (2001) Tumor hypoxia: definitions and current clinical, biologic, and molecular aspects. J Natl Cancer Inst 93(4):266–276. 10.1093/jnci/93.4.266 11181773 10.1093/jnci/93.4.266

[CR23] Jain P, Bhatia S, Thompson EW et al (2022) Population dynamics of epithelial-mesenchymal heterogeneity in cancer cells. Biomolecules 12(3):348. 10.3390/biom12030348 35327538 10.3390/biom12030348 PMC8945776

[CR24] Jiang M, Jia K, Wang L et al (2020) Alterations of DNA damage repair in cancer: from mechanisms to applications. Ann Transl Med 8(24):1685. 10.21037/atm-20-2920 33490197 10.21037/atm-20-2920 PMC7812211

[CR25] Jiménez-Sánchez J, Martínez-Rubio Á, Popov A et al (2021) A mesoscopic simulator to uncover heterogeneity and evolutionary dynamics in tumors. PLoS Comput Biol 17(2):e1008266. 10.1371/journal.pcbi.1008266 33566821 10.1371/journal.pcbi.1008266 PMC7901744

[CR26] Kawai T, Matsuo M, Takakusagi Y et al (2022) Continuous monitoring of postirradiation reoxygenation and cycling hypoxia using electron paramagnetic resonance imaging. NMR Biomed 35(10):e4783. 10.1002/nbm.4783 35661282 10.1002/nbm.4783 PMC9482554

[CR27] Kim MH, Green SD, Lin CC et al (2021) Engineering tools for regulating hypoxia in tumour models. J Cell Mol Med 25(16):7581–7592. 10.1111/jcmm.16759 34213838 10.1111/jcmm.16759 PMC8358887

[CR28] Leedale J, Herrmann A, Bagnall J et al (2014) Modeling the dynamics of hypoxia inducible factor-1 (HIF-1) within single cells and 3D cell culture systems. Math Biosci 258:33–43. 10.1016/j.mbs.2014.09.007 25245610 10.1016/j.mbs.2014.09.007

[CR29] Liu J, Hormuth DAII, Davis T et al (2021) A time-resolved experimental-mathematical model for predicting the response of glioma cells to single-dose radiation therapy. Integr Biol 13(7):167–183. 10.1093/intbio/zyab010 10.1093/intbio/zyab010 PMC8271006 34060613

[CR30] Lorenzi T, Painter KJ (2022) Trade-offs between chemotaxis and proliferation shape the phenotypic structuring of invading waves. Int J Non-Linear Mech 139:103885. 10.1016/j.ijnonlinmec.2021.103885

[CR31] Matsumoto S, Yasui H, Mitchell JB et al (2010) Imaging cycling tumor hypoxia. Can Res 70(24):10019–10023. 10.1158/0008-5472.CAN-10-2821 10.1158/0008-5472.CAN-10-2821 PMC3059188 21159626

[CR32] Matthews HK, Bertoli C, de Bruin RAM (2022) Cell cycle control in cancer. Nat Rev Mol Cell Biol 23(1):74–88. 10.1038/s41580-021-00404-3 34508254 10.1038/s41580-021-00404-3

[CR33] McKeown SR (2014) Defining normoxia, physoxia and hypoxia in tumours-implications for treatment response. Br J Radiol 87(1035):20130676. 10.1259/bjr.20130676 24588669 10.1259/bjr.20130676 PMC4064601

[CR34] Michiels C, Tellier C, Feron O (2016) Cycling hypoxia: a key feature of the tumor microenvironment. Biochimica et Biophysica Acta (BBA) Rev Cancer 1866(1):76–86. 10.1016/j.bbcan.2016.06.004 10.1016/j.bbcan.2016.06.004 27343712

[CR35] Murphy RJ, Gunasingh G, Haass NK et al (2023) Growth and adaptation mechanisms of tumour spheroids with time-dependent oxygen availability. PLoS Comput Biol 19(1):e1010833. 10.1371/journal.pcbi.1010833 36634128 10.1371/journal.pcbi.1010833 PMC9876349

[CR36] Ng N, Purshouse K, Foskolou IP et al (2018) Challenges to DNA replication in hypoxic conditions. FEBS J 285(9):1563–1571. 10.1111/FEBS.14377 29288533 10.1111/febs.14377

[CR37] Olcina M, Lecane PS, Hammond EM (2010) Targeting hypoxic cells through the DNA damage response. Clin Cancer Res 16(23):5624–5629. 10.1158/1078-0432.CCR-10-0286 20876254 10.1158/1078-0432.CCR-10-0286 PMC3000384

[CR38] Pires IM, Bencokova Z, McGurk C et al (2010) Exposure to acute hypoxia induces a transient DNA damage response which includes Chk1 and TLK1. Cell Cycle 9(13):2502. 10.4161/CC.9.13.12059 20581459 10.4161/cc.9.13.12059 PMC3040847

[CR39] Pires IM, Bencokova Z, Milani M et al (2010) Effects of acute versus chronic hypoxia on DNA damage responses and genomic instability. Can Res 70(3):925–935. 10.1158/0008-5472.CAN-09-2715 10.1158/0008-5472.CAN-09-2715 PMC2923514 20103649

[CR40] Pérez-Aliacar M, Ayensa-Jiménez J, Doblaré M (2023) Modelling cell adaptation using internal variables: accounting for cell plasticity in continuum mathematical biology. Comput Biol Med 164:107291. 10.1016/j.compbiomed.2023.107291 37586203 10.1016/j.compbiomed.2023.107291

[CR41] Qiu GZ, Jin MZ, Dai JX et al (2017) Reprogramming of the tumor in the hypoxic niche: the emerging concept and associated therapeutic strategies. Trends Pharmacol Sci 38(8):669–686. 10.1016/j.tips.2017.05.002 28602395 10.1016/j.tips.2017.05.002

[CR42] Ron A, Deán-Ben XL, Gottschalk S et al (2019) Volumetric optoacoustic imaging unveils high-resolution patterns of acute and cyclic hypoxia in a murine model of breast cancer. Can Res 79(18):4767–4775. 10.1158/0008-5472.CAN-18-3769 10.1158/0008-5472.CAN-18-3769 31097477

[CR43] Saxena K, Jolly MK (2019) Acute vs chronic vs cyclic hypoxia: their differential dynamics, molecular mechanisms, and effects on tumor progression. Biomolecules 9(8):339. 10.3390/biom9080339 31382593 10.3390/biom9080339 PMC6722594

[CR44] Stillman B (2013) Deoxynucleoside triphosphate (dNTP) synthesis and destruction regulate the replication of both cell and virus genomes. Proc Natl Acad Sci 110(35):14120–14121. 10.1073/pnas.1312901110 23946423 10.1073/pnas.1312901110 PMC3761580

[CR45] Tomasin R, Bruni-Cardoso A (2022) The role of cellular quiescence in cancer - beyond a quiet passenger. J Cell Sci 135(15):jcs259676. 10.1242/jcs.259676 35929545 10.1242/jcs.259676

[CR46] Vedel S, Nunns H, Košmrlj A et al (2016) Asymmetric damage segregation constitutes an emergent population-level stress response. Cell Syst 3(2):187–198. 10.1016/j.cels.2016.06.008 27426983 10.1016/j.cels.2016.06.008

[CR47] Viner-Breuer R, Yilmaz A, Benvenisty N et al (2019) The essentiality landscape of cell cycle related genes in human pluripotent and cancer cells. Cell Div 14(1):15. 10.1186/s13008-019-0058-4 31889988 10.1186/s13008-019-0058-4 PMC6927170

[CR48] Webb GF (1987) An operator-theoretic formulation of asynchronous exponential growth. Trans Am Math Soc 303(2):751. 10.2307/2000695

[CR49] Wells A, Griffith L, Wells JZ et al (2013) The dormancy dilemma: quiescence versus balanced proliferation. Can Res 73(13):3811–3816. 10.1158/0008-5472.CAN-13-0356 10.1158/0008-5472.CAN-13-0356 PMC3702639 23794703

[CR50] Wu C, Shi W, Zhang S (2023) ZEB1 promotes DNA homologous recombination repair and contributes to the 5-fluorouracil resistance in colorectal cancer. Am J Cancer Res 13(9):4101–4114 37818077 PMC10560938

[CR51] Xing M, Zhang F, Liao H et al (2020) Replication stress induces ATR/CHK1-dependent nonrandom segregation of damaged chromosomes. Mol Cell 78(4):714-724.e5. 10.1016/j.molcel.2020.04.005 32353258 10.1016/j.molcel.2020.04.005

[CR52] Zhang C, Cao S, Xu Y (2014) Population dynamics inside cancer biomass driven by repeated hypoxia-reoxygenation cycles. Quant Biol 2(3):85–99. 10.1007/s40484-014-0032-8

